# The inference of HIV-1 transmission direction between a man who has sex with men and his heterosexual wife based on the sequences of HIV-1 quasi-species

**DOI:** 10.1080/22221751.2021.1938693

**Published:** 2021-06-17

**Authors:** Zehua Zhou, Ping Ma, Yi Feng, Weidong Ou, Min Wei, Yiming Shao

**Affiliations:** a School of Medicine, Nankai University, Tianjin, People’s Republic of China; b Nankai University Second People’s Hospital, School of Medicine, Nankai University, Tianjin, People’s Republic of China; c National Center for AIDS/STD Control and Prevention, Chinese Center for Disease Control and Prevention, Beijing, People’s Republic of China

**Keywords:** HIV-1, MSM, marriage, transmission relationship, single-genome sequencing

## Abstract

Currently, homosexual transmission has become one of the main routes of HIV-1 spread in China. Furthermore, about 80% Chinese men, who have sex with men (MSM), feel forced to enter eventually into heterosexual marriages due to the Chinese traditional marriage culture, which may cause HIV-1 infection in families. In this study, we identified HIV-1 transmission in a family and the direction of HIV-1 transmission from a MSM to his wife and infant, which indicated Chinese MSM may have become a potential bridge of HIV-1 transmission to their wives and children. Therefore, we need to develop more effective defence measures to prevent the spread of HIV-1 in MSM families in China.

## Introduction

In some western countries, there is a more open attitude towards homosexuality, with some approved laws to legalize homosexual marriage. Men who have sex with men (MSM), therefore, have greater individual freedom of choice and legal protection [1,2]. In contrast, homosexual marriage is illegal in China and in many other countries. Therefore, more than 80% Chinese MSM will marry heterosexual women eventually [3]. In China, homosexual orientation is considered not only abnormal, but also highly discriminated, as it violates Chinese traditional culture. Traditional marriage is a social, cultural and familial obligation. Thus, to marry a heterosexual woman is the best choice for MSM to avoid social discrimination. Nowadays, HIV epidemic among MSM is a serious problem in China, because homosexual exposure has become the primary mode of HIV transmission [4–7]. The rate of homosexual transmission in MSM among HIV new infections each year in China, increased from 2.5% in 2006 to 25.5% in 2017 [8,9]. According to a survey of MSM in the United States, married MSM are more likely to have unsafe sex than unmarried MSM [10]. Furthermore, procreation is considered the main purpose of sex and marriage, which makes condom useless between couples. Therefore, MSM expose their wives to a higher risk of HIV infection.

It is important to understand the mode of HIV-1 transmission and transmission between MSM and their wives, to take effective measures to prevent and control the spread of HIV-1. Phylogenetic analysis has been used to identify the pattern of HIV-1 transmission between the suspected transmission relationship because paraphilia relationship was found between the donor’s sequences and the recipient’s sequences in the phylogenetic tree [11–14]. There are three ways of HIV-1 transmission between two known transmission HIV-positive pairs: a direct transmission, a transmission through an intermediary, or two HIV-positive individuals sharing a common infection source [15]. Therefore, HIV phylogenies were classified into three types: polyphyletic-polyphyletic (PP), paraphyletic-monophyletic (PM), and monophyletic-monophyletic (MM) patterns [16,17]. PP and PM phylogenies probably indicate direct transmission between transmission pairs, while MM phylogenies are not indicative of transmission direction [17].

In China, most MSM engage in high-risk behaviours, such as multiple sexual partners and unprotected anal intercourse, which may cause HIV-1 dual infection [18–20]. An article also showed the relatively high frequency of dual infection in Chinese MSM [21]. Standard Sanger sequencing has been applied widely to investigate HIV-1 gene PCR products. However, Sanger sequencing cannot detect variants with less than 20% of the viruses in a sample, which means that its readout only represents a consensus sequence [22,23]. Sanger sequencing always misses the sequences of minor viruses. In contrast, single-genome amplification (SGA) is based on serial dilution of a viral genome, and the dilution yields as low as 30% positive reactions for subsequent Sanger sequencing. SGA has an 80% probability of deriving from a single amplifiable template. SGA can obtain more viral variants in a sample than Sanger sequencing alone, and SGA promotes more effective analyses for the transmission of HIV-1 and the genetic characterization of viruses [24].

In this study, we applied SGA to infer the direction of transmission between a special man who has sex with men to his wife and infant. Furthermore, we studied the evolution of HIV-1 *env*, *pol* regions in the man who was superinfected for 12 months.

## Materials and methods

### Ethics statement

The participants provided written informed consent for their information. Their clinical samples were stored and used for research. Ethical approval for this study was obtained from Research Ethics Community of School of Medicine, Nankai University.

### Study participants

The study enrolled an HIV-positive family, a pair of couple and their baby, whose family were diagnosed as HIV positive in March 2017. Patients were followed up at the Department of Infectious Diseases of the Nankai University Second People’s Hospital in Tianjing, China. Clinical information was collected from the hospital electronic database and was anonymised prior to analysis. This information included age of the participant, time of HIV diagnosis, time of blood sampling, viral load and CD4^+^ T-cell count at sampling. They have signed the ethics-approved informed consent form of Nankai University.

### DNA extraction, PCR and sequencing

Whole blood samples were collected using sterile ethylenediaminetetra-acetic acid (EDTA) tubes. Provirus DNA was extracted from 200 μL of whole blood samples using QIAamp Viral DNA Mini Kit (Qiagen 51304). HIV-1 segments of *pol*, *gag*, *env* were amplified using nested polymerase chain reaction (PCR). The first PCR reaction was performed using the TaKaRa La Taq (TaKaRa Biotechnology Co. Ltd., Dalian, China) with outer primer pair F1a 5′-TGAARGAITGYACTGARAGRCAGGCTAAT-3′ (HXB2 positions 2057-2085)/F1b 5′-ACTGARAGRCAGGCTAATTTTTTAG-3 (HXB2 positions 2068-2092)/RT-R1 5′-ATCCCTGCATAAATCTGACTTGC-3′ (HXB2 positions 3370-3348), 44F 5′-ACAGTRCARTGYACACATGG-3′ (HXB2 positions 6954-6978)/35R 5′-CACTTCTCCAATTGTCCITCA-3′ (HXB2 positions 7648-7668) and GAG-L 5′-TCGACGCAGGACTCGGCTTGC-3′ (HXB2 positions 686-707)/GAG-E2 5′-TCCAACAGCCCTTTTTCCTAGG-3′ (HXB2 positions 2011-2032) to amplify *pol*, *env*, and *gag* region of HIV-1, respectively. The second PCR reactions were performed using the TaKaRa La Taq with inner primer pair PRT-F2 5′- CTTTARCTTCCCTCARATCACTCT -3′ (HXB2 positions 2243-2266)/RT-R2 5′- CTTCTGTATGTCATTGACAGTCC -3′ (HXB2 positions 3326-3304), DR7m4 5′-TGTAAAACGACGGCCAGTCTGTTAAATGGYAGYCTAGC-3′ (HXB2 positions 7002-7021)/DR8m4 5′-CAGGAAACAGGCTATGACCCTCCAATTGTYCCTCATAT-3′ and GUX 5′-AGGAGAGAGATGGGTGCGAGAGCGTC-3′ (HXB2 positions 781-806)/GDX 5′-GGCTAGTTCCTCCTACTCCCTGACAT-3′ (HXB2 positions1836-1861) to amplify *env*, *gag*, and *pol* region of HIV-1, respectively. Amplified PCR products were separated on an agarose gel, and then cut, purified and sent for sequencing by Beijing Tianyi Huiyuan BioTechnologies Co., Ltd.

### Single genome amplification

Single genome amplification (SGA) was performed, as previously described [25]. To amplify HIV-1 *pol* and *env* genes, the first round PCR was performed using *pol* gene primers F1a/RT-R1 and *env* gene primer 44F/5R. The second round PCR was performed with *pol* gene primers PRT-F2/ RT-R2 and *env* gene primer DR7m4/DR8m4. The *pol* and *env* regions of PCR products were directly sequenced by the cycle sequencing and dye terminator methods. Individual sequences were assembled and edited by Sequencher 4.7 (Gene Codes).

### Phylogenetic linkage analysis of couple sequences

Maximum-likelihood trees of *pol* and *env* sequences were constructed using a general time reversible (GTR) nucleotide substitution with a gamma distribution of rates, based on 1000 bootstrap replicates. Using BLAST programme in HIV database, we identified the most matching sequences, which were added as control sequences in phylogenetic analysis. Some unrelated sequences isolated from local patients (7 *pol* seq and 6 *env* seq) and reference sequences reported in a previous study [26] also were regarded as controls to ensure the real relationship in clusters. Cluster bootstrap value ≥80% was considered as a genetic linkage.

## Results

### Patients’ characteristics

The date of diagnosis of the couple and their baby was on March 3, 2017. The man and his wife were both 25 years old, and their kid was about 4 months old. The number of viral loads in the kid was 902,464 HIV RNA copies/ml. CD4^+^ cell count of the man and woman was 971 cells/μl and 327cells/μl, respectively.

### The phylogenetic analyses of patient’s sequences obtained by Sanger sequencing showed there was no transmission relationship between the couple

**Figure 1.**
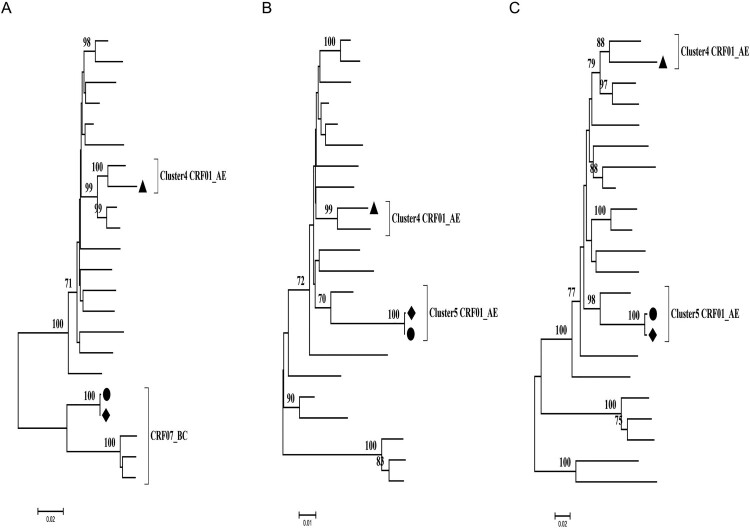
The Maximum-likelihood tree of *gag*, *pol*, *env* sequences of the couple and their kid. The kid, woman and man are marked black circle •, diamond ♦ and triangle ▴, respectively. (A) The Maximum-likelihood tree of *gag*; (B) The Maximum-likelihood tree of *pol*; (C) The Maximum-likelihood tree of *env*.

HIV-1 *pol*, *gag*, and *env* genes of the couple and kid were successfully amplified and then sequenced by Sanger sequencing. The phylogenetic trees of the conserved *pol*, *gag* and highly variable *env* C2V5 genes were constructed for identifying HIV-1 subtypes and transmission relationship. The man was infected with g4 CRF01_AE (Figure 1). The woman and her baby were likely to be infected with a recombinant virus comprising the genes of g5 CRF01_AE and CRF07_BC, because the subtype of *gag* gene was CRF07_BC (Figure 1(A)), but *pol* and *env* genes were g5 CRF01_AE (Figure 1(B,C)). Phylogenetic analyses showed the sequence of woman and her baby was closely related and clustered in an exclusive branch, but the sequence of the man was separated into a different branch, which indicates a non-genetic linkage between the couple.

### The couple and their kid were infected with different HIV-1 recombinant strains

**Figure 2.**
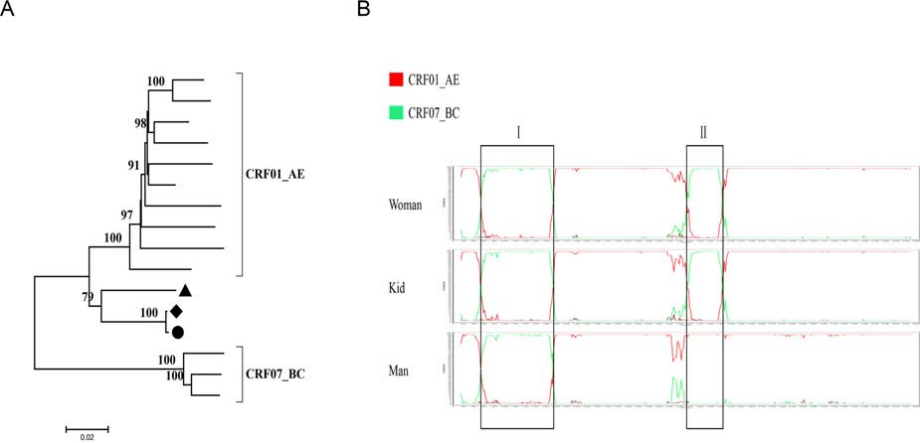
The analysis of full-length genome of patients through the phylogenetic tree and Simplot software. The kid, woman and man are marked black circle, diamond and triangle, respectively. (A) the Maximum-likelihood tree of full-length genome of the couple and their kid; (B) the breakpoint analysis of the couple and their kid.

HIV Near Full-length genome (NFLG) sequences of patients were obtained by nested PCR. The NFLG sequences of this study have been directly deposited into GenBank as MK634699, MH220193 and MH220194. The Maximum-likelihood tree of full-length genome showed they were related closely and clustered in the exclusive branch and the viruses indicated they may be infected with a recombinant virus (Figure 2(A)). The analysis of breakpoints in the full-length genome of the couple and their kid reflected they were infected with CRF01_AE/CRF07_BC recombinant viruses, but full-length genome of the woman and kid indicated that one more region of CRF07_BC was inserted into the backbone of CRF01_AE. We found the sites of the recombination breakpoints of 1160–2590 (relative to HXB2) in three strains, but 5187–5682 (relative to HXB2) only in two strains from the women and kid. Therefore, we suspected dual infection occurred in the woman and kid (Figure 2(B)).

### HIV dual infection in the man

**Figure 3.**
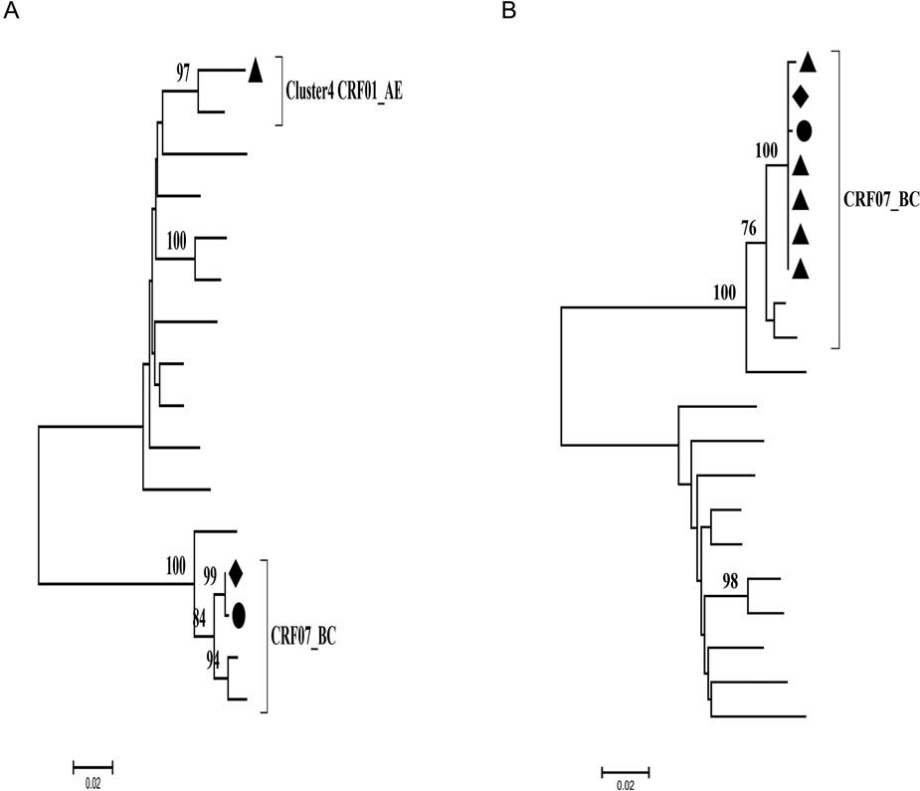
The phylogenetic analysis of vif sequences obtained by Sanger sequencing and Single-genome amplification (SGA). The kid, woman and man are marked black circle, diamond and triangle, respectively. (A) The Maximum-likelihood tree of *vif* gene obtained by Sanger sequencing; (B) We obtained *vif* sequences from the man through SGA, and combined with the *vif* sequences of the woman and kid sequenced by Sanger sequencing to construct the Maximum-likelihood tree.

The Maximum-likelihood tree of the fragment of CRF07_BC, which was inserted into *vif* and *vpr* gene of g5 CRF01_AE in the recombinant virus of the woman and kid and the consistent region in the virus of the man, showed they were infected with different viruses (Figure 3(A)). This conclusion is contradictory to the result of phylogenetic analysis of full-length genome (Figure 3(A)). For elucidating this question, we obtained more sequences of that region in the man through SGA to construct the Maximum-likelihood tree again. The result indicated there was the same fragment of CRF07_BC in the man, which suggests that the recombination of HIV-1 between two different strains may occur in the man (Figure 3(B)).

The phylogenetic analysis of the couple’s *pol*, *env* sequences obtained by SGA identified the transmission relationship between the couple.

**Figure 4.**
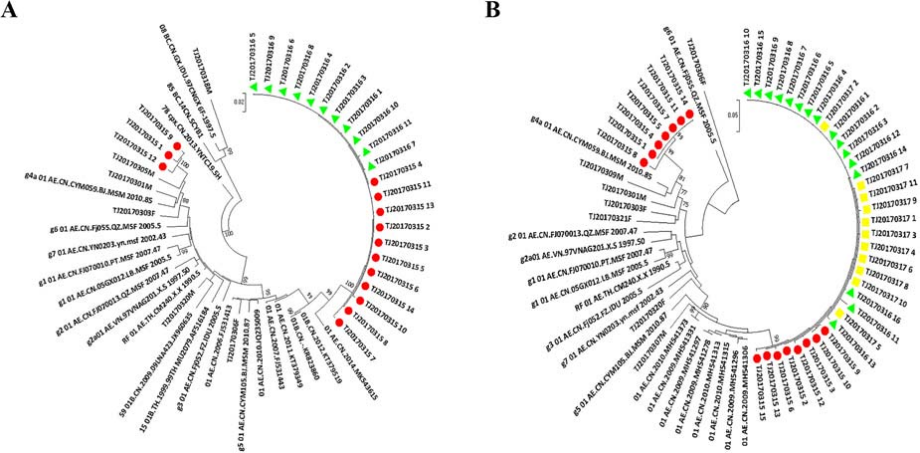
Maximum-likelihood trees of pol and env sequences of the couple and kid using BLAST-selected GenBank and local controls in A and B, respectively. The man, woman and kid are marked red circle, green triangle and yellow square, respectively. The black represents control sequences. (A) Maximum-likelihood tree of *pol* sequences; (B) Maximum-likelihood tree of *env* sequences.

Quasi-species sequences of *pol*, *env* in the man were obtained by SGA. The Maximum-likelihood tree of *pol* sequences showed the man was infected with HIV-1 g5 CRF01_AE and g4 CRF01_AE (Figure 4(A)), either of the two was also detected in the woman and kid. Moreover, paraphyletic-monophyletic is observed in this phylogenetic tree. The Maximum-likelihood tree of *env* sequences showed the man also was infected with g5 CRF01_AE and g4 CRF01_AE (Figure 4(B)). The phylogenetic relationship between the couple is in accordance with in *env* and *pol*, which indicates the direction of transmission from the man, who has sex with the men, to his wife and kid.

### Evolution of HIV-1 quasi-species overtime.

**Figure 5.**
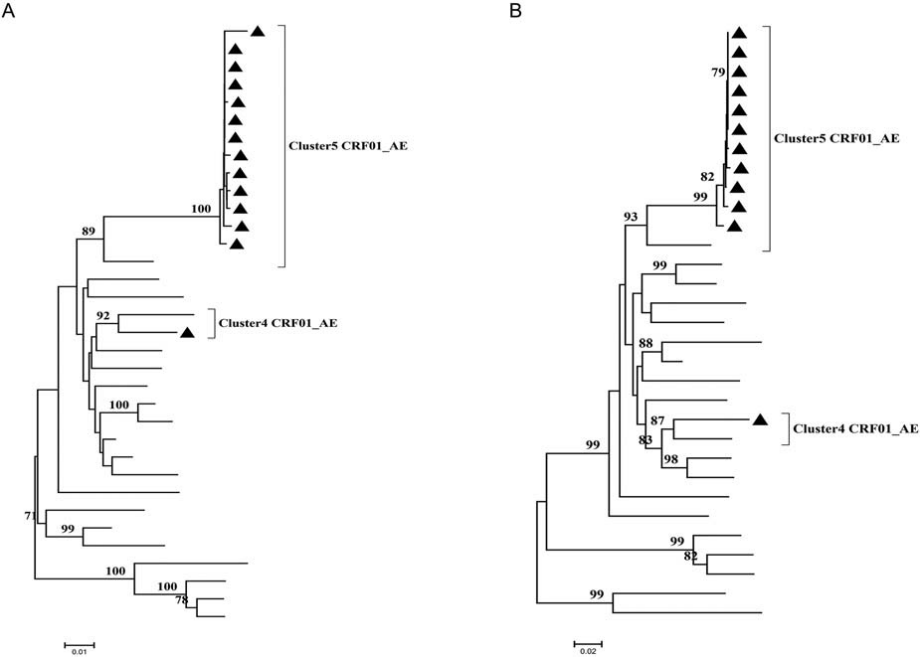
The phylogenetic analysis of *pol*, *env* sequences obtained by SGA from the second whole blood sample of the man. The man is marked black triangle ▴. (A) The Maximum-likelihood tree of *pol* sequences; (B) The Maximum-likelihood tree of *env* sequence.

We resampled the whole blood of the man after one year of the first sampling. The *pol*, *env* sequences obtained by SGA were constructed phylogenetic trees. The Maximum-likelihood tree of *pol* sequences showed the rate of g4 CRF01_AE variants compared with the first results of SGA decreased to low frequency (Figure 5(A)), and the similar rate also was reflected in the Maximum-likelihood tree of *env* sequences (Figure 5 (B)).

## Discussion

In this study, we inferred the direction of HIV-1 transmission in the couple and the direction of HIV transmission is from the man to the woman and the baby.

In the previous report, in non-disclosed men who have sex with men (MSM) population in developing countries, self-reported only heterosexual men became infected through homosexual contacts [27]. In some developing countries like China, homosexuality, however, is regarded as stigma, which make MSM feel fear to disclose their sex orientation. Therefore, the majority of Chinese MSM eventually get married to heterosexual women for escaping the pressure of society and family. MSM became the bridge of the HIV transmission from high-risk population to general population in China. According to newly diagnosed cases of HIV/AIDS per year, the heterosexual transmission has reached over 60% among all transmissions in China since 2011 [28], and some cases may be transmitted by their husbands who belong to MSM population. Moreover, HIV-1 positive women, who were infected by MSM, may transmit HIV to their infants. In this study, we identified the HIV-1 transmission direction from an MSM to his wife and kid. Therefore, the HIV-1 infection rate caused by MSM population was underestimated. Now, some strategies were taken to prevent and control the prevalence of HIV-1 among heterosexual and homosexual transmission, but ignored the population who are MSM and get married to women. Therefore, we need to take more effective strategies to control HIV-1 infection.

Some epidemiological studies showed the most Chinese MSM engage in high-risk behaviour, such as multiple partners and unprotected anal intercourse, which might increase opportunities for HIV-1 dual infection [18–20]. In the United States, dual infection among MSM was about 14.4% [29]. The occurrence of dual infection among Chinese MSM was higher than that of the United States [21], which means active high-risk behaviour in the Chinese MSM population. In this study, the man was infected with two different HIV-1 strains, and either of two was detected in his wife and kid (Figure 4(A,B)). We observed that the phylogenetic analysis of sequences, obtained by different sequencing approaches, Sanger sequencing only or SGA, resulted in a significant different epidemiological link analysis and transmission relationship between the couple. The Maximum-likelihood tree of *gag*, *pol*, *env* sequences obtained by Sanger sequencing showed a non-genetic linkage between the couple (Figure 1), but a clear transmission relationship between the couple through SGA (Figure 4). The sensitivity limitation of population-based sequencing like Sanger sequencing is difficult to detected minor viral populations (≦20%), which may reflect inaccurately the occurrence of dual infection in the host. Therefore, more sensible sequencing methods like SGA should be applied for constructing phylogenetic analyses to understand the dynamic of HIV-1 transmission accurately.

Our result is consistent with some published reports that dual infection can increase genetic diversity within the host [30], but only either of the two seems to be transmitted by sexual contact [31]. The man was infected with two different strains, but only either of the two can be detected in his wife (Figure 4). In the meantime, we observed the generation of a new recombinant form by two different subtype strains in the man (Figure 2(B)). The occurrence of recombination between two different strains may facilitate immune evasion, development of drug resistance and disease progression [32,33]. Therefore, the tracing of HIV-1 infection in the host is important for understanding the transmission and evolution of the virus. After one year of follow-up, we recollected the whole blood of the man. The Maximum-likelihood trees of *pol*, *env* sequences showed the change of the ratio of cluster4 CRF01_AE to cluster5 CRF01_AE compared with the first results, and cluster5 CRF01_AE turned into the predominant strain (Figure 5(A,B)). Some previous articles reported that the most common fate of the secondary infecting strain initially becomes nearly as frequent as the first infecting strain, but then tends to disappear rapidly [34]. Our results also showed either of two different strains tends to disappear, the other became the predominant strain over the time.

There were several limitations to this study. First, only a case of the HIV transmission form a man who has sex with men to his heterosexual wife inaccurately reflected the pattern of HIV-1 transmission between MSM and their wives. Therefore, we hope more samples can be enrolled into our study. Secondly, the whole blood samples of the man were just collected twice, and the second sample was sampled at an interval of one year. Therefore, the results may not elucidate precisely the influence of dual infection on the evolution of viruses.

In summary, we identified the HIV-1 transmission direction from a man who has sex with men to his wife and kid based on the SGA method. Moreover, we found the man was infected with two different strains and developed a recombinant form between two strains, and it finally became the predominant virus. Our results showed dual infection can affect the judgment of the transmission relationship. Therefore, more sensible sequencing methods are needed to construct the phylogenetic tree for inferring the direction of transmission accurately. We need to take urgent measures to make more effective HIV prevention measure appropriate for MSM who get married to women.

## Supplementary Material

sequences_information.doc
