# Supplementary material for: The inference of HIV-1 transmission direction between a man who has sex with men and his heterosexual wife based on the sequences of HIV-1 quasi-species
Source: Emerg Microbes Infect. 2021 Jun 17;10(1):1209–16. doi: 10.1080/22221751.2021.1938693 (PMC8676586; doi:10.1080/22221751.2021.1938693)
Supplement: sequences_information.doc [file TEMI_A_1938693_SM2376.doc]

Supplementary:

1.*Pol* SGA sequences of the couple

>TJ20170316_1

------ATCACTCTTTGGCAACGACCCCTTGTTGCCATAAGGATAGGGGGGCAAGTAAAG

GAAGCTCTACTAGATACAGGAGCAGATGATACAGTATTAGAAGACATAAATTTGCCAGGG

AAATGGAAACCAAAAATGATAGGGGGAATTGGAGGTTTTATCAAAGTAAGACAGTATGAA

CAGGTACCCATAGAAATCTGTGGACATAAAACTATAGGTACAGTGTTAATAGGACCTACA

CCTGTCAACATAATTGGGAGAAATCTGTTGACGCAGCTTGGTTGTACTTTAAATTTTCCA

ATCAGTCCTATTGACACTGTACCAGTAAAATTAAAGCCAGGAATGGATGGACCAAAGGTT

AAACAATGGCCATTAACAGAAGAAAAAATAAAAGCATTAATAGAAATTTGTAAAGAGATG

GAAGAGGAAGGAAAAATCTCAAAAATTGGGCCTGAAAATCCTTACAATACTCCAGTATTT

GCTATAAGGAAAAAGGACAGCACCAAATGGAGGAAATTAGTAGATTTCAGAGAGCTCAAT

AAAAGAACTCAGGACTTTTGGGAAGTTCAATTAGGAATACCGCATCCAGCAGGTTTAAAG

AAAAAGAAATCAGTAACAGTACTAGATGTGGGAGATGCATATTTCTCAGTTCCTTTAGAT

AAAAGCTTTAGAAAGTATACTGCATTTACCATACCTAGTACAAACAATGAGACACCAGGA

ATCAGATATCAGTACAATGTGCTGCCACAGGGATGGAAAGGATCACCGGCAATATTCCAG

AGTAGCATGACAAAGATCTTAGAACCTTTTAGAATAAAAAATCCAGAAATAGTCATCTAT

CAATATATGGATGACTTGTATGTAGGCTCCGATTTAGAAATAGGGCAGCACAGAATAAAA

ATAGAGGAGCTGAGAGCTCATCTATTGAGCTGGGGACTTACTACCCCAGACAAAAAGCAT

CAGAAGGAACCTCCATTCCTTTGGATGGGATACGAACTCCATCCTGACAGATGGACAGTC

CAGCCAATAAAACTGCCAGAAAAAGACAGCTGGACTGTCAATG

>TJ20170316_2

-----GATCACTCTTTGGCAACGACCCCTTGTTGCCATAAGGATAGGGGGGCAAGTAAAG

GAAGCTCTACTAGATACAGGAGCAGATGATACAGTATTAGAAGACATAAATTTGCCAGGG

AAATGGAAACCAAAAATGATAGGGGGAATTGGAGGTTTTATCAAAGTAAGACAGTATGAA

CAGGTACCCATAGAAATCTGTGGACATAAAACTATAGGTACAGTGTTAATAGGACCTACA

CCTGTCAACATAATTGGGAGAAATCTGTTGACTCAGCTTGGTTGTACTTTAAATTTTCCA

ATCAGTCCTATTGACACTGTACCAGTAAAATTAAAGCCAGGAATGGATGGACCAAAGGTT

AAACAATGGCCATTAACAGAAGAAAAAATAAAAGCATTAATAGAAATTTGTAAAGAGATG

GAAGAGGAAGGAAAAATCTCAAAAATTGGGCCTGAAAATCCTTACAATACTCCAGTATTT

GCTATAAGGAAAAAGGACAGCACCAAATGGAGGAAATTAGTAGATTTCAGAGAGCTCAAT

AAAAGAACTCAGGACTTTTGGGAAGTTCAATTAGGAATACCGCATCCAGCAGGTTTAAAG

AAAAAGAAATCAGTAACAGTACTAGATGTGGGAGATGCATATTTCTCAGTTCCTTTAGAT

AAAAGCTTTAGAAAGTATACTGCATTTACCATACCTAGTACAAACAATGAGACACCAGGA

ATCAGATATCAGTACAATGTGCTGCCACAGGGATGGAAAGGATCACCGGCAATATTCCAG

AGTAGCATGACAAAGATCTTAGAACCTTTTAGAATAAAAAATCCAGAAATAGTCATCTAT

CAATATATGGATGACTTGTATGTAGGCTCCGATTTAGAAATAGGGCAGCACAGAATAAAA

ATAGAGGAGCTGAGAGCTCATCTATTGAGCTGGGGACTTACTACCCCAGACAAAAAGCAT

CAGAAGGAACCTCCATTCCTTTGGATGGGATACGAACTCCATCCTGACAGATGGACAGTC

CAGCCAATAAAACTGCCAGAAAAAGACAG--------------

>TJ20170316_3

-----GATCACTCTTTGGCAACGACCCCTTGTTGCCATAAGGATAGGGGGGCAAGTAAAG

GAAGCTCTACTAGATACAGGAGCAGATGATACAGTATTAGAAGACATAAATTTGCCAGGG

AAATGGAAACCAAAAATGATAGGGGGAATTGGAGGTTTTATCAAAGTAAGACAGTATGAA

CAGGTACCCATAGAAATCTGTGGACATAAAACTATAGGTACAGTGTTAATAGGACCTACA

CCTGTCAACATAATTGGGAGAAATCTGTTGACTCAGCTTGGTTGTACTTTAAATTTTCCA

ATCAGTCCTATTGACACTGTACCAGTAAAATTAAAGCCAGGAATGGATGGACCAAAGGTT

AAACAATGGCCATTAACAGAAGAAAAAATAAAAGCATTAATAGAAATTTGTAAAGAGATG

GAAGAGGAAGGAAAAATCTCAAAAATTGGGCCTGAAAATCCTTACAATACTCCAGTATTT

GCTATAAGGAAAAAGGACAGCACCAAATGGAGGAAATTAGTAGATTTCAGAGAGCTCAAT

AAAAGAACTCAGGACTTTTGGGAAGTTCAATTAGGAATACCGCATCCAGCAGGTTTAAAG

AAAAAGAAATCAGTAACAGTACTAGATGTGGGAGATGCATATTTCTCAGTTCCTTTAGAT

AAAAGCTTTAGAAAGTATACTGCATTTACCATACCTAGTACAAACAATGAGACACCAGGA

ATCAGATATCAGTACAATGTGCTGCCACAGGGATGGAAAGGATCACCGGCAATATTCCAG

AGTAGCATGACAAAGATCTTAGAACCTTTTAGAATAAAAAATCCAGAAATAGTCATCTAT

CAATATATGGATGACTTGTATGTAGGCTCCGATTTAGAAATAGGGCAGCACAGAATAAAA

ATAGAGGAGCTGAGAGCTCATCTATTGAGCTGGGGACTTACTACCCCAGACAAAAAGCAT

CAGAAGGAACCTCCATTCCTTTGGATGGGATACGAACTCCATCCTGACAGATGGACAGTC

CAGCCAATAAAACTGCCAGAAAAAGACAGCTG-----------

>TJ20170316_4

-------TCACTCTTTGGCAACGACCCCTTGTTGCCATAAGGATAGGGGGGCAAGTAAAG

GAAGCTCTACTAGATACAGGAGCAGATGATACAGTATTAGAAGACATAAATTTGCCAGGG

AAATGGAAACCAAAAATGATAGGGGGAATTGGAGGTTTTATCAAAGTAAGACAGTATGAA

CAGGTACCCATAGAAATCTGTGGACATAAAACTATAGGTACAGTGTTAATAGGACCTACA

CCTGTCAACATAATTGGGAGAAATCTGTTGACTCAGCTTGGTTGTACTTTAAATTTTCCA

ATCAGTCCTATTGACACTGTACCAGTAAAATTAAAGCCAGGAATGGATGGACCAAAGGTT

AAACAATGGCCATTAACAGAAGAAAAAATAAAAGCATTAATAGAAATTTGTAAAGAGATG

GAAGAGGAAGGAAAAATCTCAAAAATTGGGCCTGAAAATCCTTACAATACTCCAGTATTT

GCTATAAGGAAAAAGGACAGCACCAAATGGAGGAAATTAGTAGATTTCAGAGAGCTCAAT

AAAAGAACTCAGGACTTTTGGGAAGTTCAATTAGGAATACCGCATCCAGCAGGTTTAAAG

AAAAAGAAATCAGTAACAGTACTAGATGTGGGAGATGCATATTTCTCAGTTCCTTTAGAT

AAAAGCTTTAGAAAGTATACTGCATTTACCATACCTAGTACAAACAATGAGACACCAGGA

ATCAGATATCAGTACAATGTGCTGCCACAGGGATGGAAAGGATCACCGGCAATATTCCAG

AGTAGCATGACAAAGATCTTAGAACCTTTTAGAATAAAAAATCCAGAAATAGTCATCTAT

CAATATATGGATGACTTGTATGTAGGCTCCGATTTAGAAATAGGGCAGCACAGAATAAAA

ATAGAGGAGCTGAGAGCTCATCTATTGAGCTGGGGACTTACTACCCCAGACAAAAAGCAT

CAGAAGGAACCTCCATTCCTTTGGATGGGATACGAACTCCATCCTGACAGATGGACTGTC

CAGCCAATAAAACTGCCAGAAAAAGACAGCTGGACTG------

>TJ20170316_5

-------TCACTCTTTGGCAACGACCCCTTGTTGCCATAAGGATAGGGGGGCAAGTAAAG

GAAGCTTTACTAGATACAGGAGCAGATGATACAGTATTAGAAGACATAAATTTGCCAGGG

AAATGGAAACCAAAAATGATAGGGGGAATTGGAGGTTTTATCAAAGTAAGACAGTATGAA

CAGGTACCCATAGAAATCTGTGGACATAAAACTATAGGTACAGTGTTAATAGGACCTACA

CCTGTCAACATAATTGGGAGAAATCTGTTGACTCAGCTTGGTTGTACTTTAAATTTTCCA

ATCAGTCCTATTGACACTGTACCAGTAAAATTAAAGCCAGGAATGGATGGACCAAAGGTT

AAACAATGGCCATTAACAGAAGAAAAAATAAAAGCATTAATAGAAATTTGTAAAGAGATG

GAAGAGGAAGGAAAAATCTCAAAAATTGGGCCTGAAAATCCTTACAATACTCCAGTATTT

GCTATAAGGAAAAAGGACAGCACCAAATGGAGGAAATTAGTAGATTTCAGAGAGCTCAAT

AAAAGAACTCAGGACTTTTGGGAAGTTCAATTAGGAATACCGCATCCAGCAGGTTTAAAG

AAAAAGAAATCAGTAACAGTACTAGATGTGGGAGATGCATATTTCTCAGTTCCTTTAGAT

AAAAGCTTTAGAAAGTATACTGCATTTACCATACCTAGTACAAACAATGAGACACCAGGA

ATCAGATATCAGTACAATGTGCTGCCACAGGGATGGAAAGGATCACCGGCAATATTCCAG

AGTAGCATGACAAAGATCTTAGAACCTTTTAGAATAAAAAATCCAGAAATAGTCATCTAT

CAATATATGGATGACTTGTATGTAGGCTCCGATTTAGAAATAGGGCAGCACAGAATAAAA

ATAGAGGAGCTGAGAGCTCATCTATTGAGCTGGGGACTTACTACCCCAGACAAAAAGCAT

CAGAAGGAACCTCCATTCCTTTGGATGGGATACGAACTCCATCCTGACAGA---------

-------------------------------------------

>TJ20170316_6

------------TT-TGGCAACGACCCCTTGTTGCCATAAGGATAGGGGGGCAAGTAAAG

GAAGCTCTACTAGATACAGGAGCAGATGATACAGTATTAGAAGACATAAATTTGCCAGGG

AAATGGAAACCAAAAATGATAGGGGGAATTGGAGGTTTTATCAAAGTAAGACAGTATGAA

CAGGTACCCATAGAAATCTGTGGACATAAAACTATAGGTACAGTGTTAATAGGACCTACA

CCTGTCAACATAATTGGGAGAAATCTGTTGACTCAGCTTGGTTGTACTTTAAATTTTCCA

ATCAGTCCTATTGACACTGTACCAGTAAAATTAAAGCCAGGAATGGATGGACCAAAGGTT

AAACAATGGCCATTAACAGAAGAAAAAATAAAAGCATTAATAGAAATTTGTAAAGAGATG

GAAGAGGAAGGAAAAATCTCAAAAATTGGGCCTGAAAATCCTTACAATACTCCAGTATTT

GCTATAAGGAAAAAGGACAGCACCAAATGGAGGAAATTAGTAGATTTCAGAGAGCTCAAT

AAAAGAACTCAGGACTTTTGGGAAGTTCAATTAGGAATACCGCATCCAGCAGGTTTAAAG

AAAAAGAAATCAGTAACAGTACTAGATGTGGGAGATGCATATTTCTCAGTTCCTTTAGAT

AAAAGCTTTAGAAAGTATACTGCATTTACCATACCTAGTACAAACAATGAGACACCAGGA

ATCAGATATCAGTACAATGTGCTGCCACAGGGATGGAAAGGATCACCGGCAATATTCCAG

AGTAGCATGACAAAGATCTTAGAACCTTTTAGAATAAAAAATCCAGAAATAGTCATCTAT

CAATATATGGATGACTTGTATGTAGGCTCCGATTTAGAAATAGGGCAGCACAGAATAAAA

ATAGAGGAGCTGAGAGCTCATCTATTGAGCTGGGGACTTACTACCCCAGACAAAAAGCAT

CAGAAGGAACCTCCATTCCTTTGGATGGGATACGAACTCCATCCTGACAGATGGACAGTC

CAGCCAATAAAACTGCCAAAAAAAGACAGCTGGA---------

>TJ20170316_7

------ATCACTCTTTGGCAACGACCCCTTGTTGCCATAAGGATAGGGGGGCAAGTAAAG

GAAGCTCTACTAGATACAGGAGCAGATGATACAGTATTAGAAGACATAGATTTGCCAGGG

AAATGGAAACCAAAAATGATAGGGGGAATTGGAGGTTTTATCAAAGTAAGACAGTATGAA

CAGGTACCCATAGAAATCTGTGGACATAAAACTATAGGTACAGTGTTAATAGGACCTACA

CCTGTCAACATAATTGGGAGAAATCTGTTGACTCAGCTTGGTTGTACTTTAAATTTTCCA

ATCAGTCCTATTGACACTGTACCAGTAAAATTAAAGCCAGGAATGGATGGACCAAAGGTT

AAACAATGGCCATTAACAGAAGAAAAAATAAAAGCATTAATAGAAATTTGTAAAGAGATG

GAAGAGGAAGGAAAAATCTCAAAAATTGGGCCTGAAAATCCTTACAATACTCCAGTATTT

GCTATAAGGAAAAAGGACAGCACCAAATGGAGGAAATTAGTAGATTTCAGAGAGCTCAAT

AAAAGAACTCAGGACTTTTGGGAAGTTCAATTAGGAATACCGCATCCAGCAGGTTTAAAG

AAAAAGAAATCAGTAACAGTACTAGATGTGGGAGATGCATATTTCTCAGTTCCTTTAGAT

AAAAGCTTTAGAAAGTATACTGCATTTACCATACCTAGTACAAACAATGAGACACCAGGA

ATCAGATATCAGTACAATGTGCTGCCACAGGGATGGAAAGGATCACCGGCAATATTCCAG

AGTAGCATGACAAAGATCTTAGAACCTTTTAGAATAAAAAATCCAGAAATAGTCATCTAT

CAATATATGGATGACTTGTATGTAGGCTCCGATTTAGAAATAGGGCAGCACAGAATAAAA

ATAGAGGAGCTGAGAGCTCATCTATTGAGCTGGGGACTTACTACCCCAGACAAAAAGCAT

CAGAAGGAACCTCCATTCCTTTGGATGGGATACGAACTCCATCCTGACAGA---------

-------------------------------------------

>TJ20170316_8

------ATCACTCTTTGGCAACGACCCCTTGTTGCCATAAGGATAGGGGGGCAAGTAAAG

GAAGCTCTACTAGATACAGGAGCAGATGATACAGTATTAGAAGACATAAATTTGCCAGGG

AAATGGAAACCAAAAATGATAGGGGGAATTGGAGGTTTTATCAAAGTAAGACAGTATGAA

CAGGTACCCATAGAAATCTGTGGACATAAAACTATAGGTACAGTGTTAATAGGACCTACA

CCTGTCAACATAATTGGGAGAAATCTGTTGACTCAGCTTGGTTGTACTTTAAATTTTCCA

ATCAGTCCTATTGACACTGTACCAGTAAAATTAAAGCCAGGAATGGATGGACCAAAGGTT

AAACAATGGCCATTAACAGAAGAAAAAATAAAAGCATTAATAGAAATTTGTAAAGAGATG

GAAGAGGAAGGAAAAATCTCAAAAATTGGGCCTGAAAATCCTTACAATACTCCAGTATTT

GCTATAAGGAAAAAGGACAGCACCAAATGGAGGAAATTAGTAGATTTCAGAGAGCTCAAT

AAAAGAACTCAGGACTTTTGGGAAGTTCAATTAGGAATACCGCATCCAGCAGGTTTAAAG

AAAAAGAAATCAGTAACAGTACTAGATGTGGGAGATGCATATTTCTCAGTTCCTTTAGAT

AAAAGCTTTAGAAAGTATACTGCATTTACCATACCTAGTACAAACAATGAGACACCAGGA

ATCAGATATCAGTACAATGTGCTGCCACAGGGATGGAAAGGATCACCGGCAATATTCCAG

AGTAGCATGACAAAGATCTTAGAACCTTTTAGAATAAAAAATCCAGAAATAGTCATCTAT

CAATATATGGATGACTTGTATGTAGGCTCCGATTTAGAAATAGGGCAGCACAGAATAAAA

ATAGAGGAGCTGAGAGCTCATCTATTGAGCTGGGGACTTACTACCCCAGACAAAAAGCAT

CAGAAGGAACCTCCATTCCTTTGGATGGGATACGAACTCCATCCTGACAGATGGACAGTC

CAGCCAATAAAACTGCCAGAAAAAGACAGCTGGACTGTCAAT-

>TJ20170316_9

------ATCACTCTTTGGCAACGACCCCTTGTTGCCATAAGGATAGGGGGGCAAGTAAAG

GAAGCTCTACTAGATACAGGAGCAGATGATACAGTATTAGAAGACATAAATTTGCCAGGG

AAATGGAAACCAAAAATGATAGGGGGAATTGGAGGTTTTATCAAAGTAAGACAGTATGAA

CAGGTACCCATAGAAATCTGTGGACATAAAACTATAGGTACAGTGTTAATAGGACCTACA

CCTGTCAACATAATTGGGAGAAATCTGTTGACTCAGCTTGGTTGTACTTTAAATTTTCCA

ATCAGTCCTATTGACACTGTACCAGTAAAATTAAAGCCAGGAATGGATGGACCAAAGGTT

AAACAATGGCCATTAACAGAAGAAAAAATAAAAGCATTAATAGAAATTTGTAAAGAGATG

GAAGAGGAAGGAAAAATCTCAAAAATTGGGCCTGAAAATCCTTACAATACTCCAGTATTT

GCTATAAGGAAAAAGGACAGCACCAAATGGAGGAAATTAGTAGATTTCAGAGAGCTCAAT

AAAAGAACTCAGGACTTTTGGGAAGTTCAATTAGGAATACCGCATCCAGCAGGTTTAAAG

AAAAAGAAATCAGTAACAGTACTAGATGTGGGAGATGCATATTTCTCAGTTCCTTTAGAT

AAAAGCTTTAGAAAGTATACTGCATTTACCATACCTAGTACAAACAATGAGACACCAGGA

ATCAGATATCAGTACAATGTGCTGCCACAGGGATGGAAAGGATCACCGGCAATATTCCAG

AGTAGCATGACAAAGATCTTAGAACCTTTTAGAATAAAAAATCCAGAAATAGTCATCTAT

CAATATATGGATGACTTGTATGTAGGCTCCGATTTAGAAATAGGGCAGCACAGAATAAAA

ATAGAGGAGCTGAGAGCTCATCTATTGAGCTGGGGACTTACTACCCCAGACAAAAAGCAT

CAGAAGGAACCTCCATTCCTTTGGATGGGATACGAACTCCATCCTGACAGATGGACAGTC

CAGCCAATAAAACTGCCAGAAAAAGACAGCTGGACTGTCAATG

>TJ20170316_10

------------TT-TGGCAACGACCCCTTGTTGCCATAAGGATAGGGGGGCAAGTAAAG

GAAGCTCTACTAGATACAGGAGCAGATGATACAGTATTAGAAGACATAAATTTGCCAGGG

AAATGGAAACCAAAAATGATAGGGGGAATTGGAGGTTTTATCAAAGTAAGACAGTATGAA

CAGGTACCCATAGAAATCTGTGGACATAAAACTATAGGTACAGTGTTAATAGGACCTACA

CCTGTCAACATAATTGGGAGAAATCTGTTGACTCAGCTTGGTTGTACTTTAAATTTTCCA

ATCAGTCCTATTGACACTGTACCAGTAAAATTAAAGCCAGGAATGGATGGACCAAAGGTT

AAACAATGGCCATTAACAGAAGAAAAAATAAAAGCATTAATAGAAATTTGTAAAGAGATG

GAAGAGGAAGGAAAAATCTCAAAAATTGGGCCTGAAAATCCTTACAATACTCCAGTATTT

GCTATAAGGAAAAAGGACAGCACCAAATGGAGGAAATTAGTAGATTTCAGAGAGCTCAAT

AAAAGAACTCAGGACTTTTGGGAAGTTCAATTAGGAATACCGCATCCAGCAGGTTTAAAG

AAAAAGAAATCAGTAACAGTACTAGATGTGGGAGATGCATATTTCTCAGTCCCTTTAGAT

AAAAGCTTTAGAAAGTATACTGCATTTACCATACCTAGTACAAACAATGAGACACCAGGA

ATCAGATATCAGTACAATGTGCTGCCACAGGGATGGAAAGGATCACCGGCAATATTCCAG

AGTAGCATGACAAAGATCTTAGAACCTTTTAGAATAAAAAATCCAGAAATAGTCATCTAT

CAATATATGGATGACTTGTATGTAGGCTCCGATTTAGAAATAGGGCAGCACAGAATAAAA

ATAGAGGAGCTGAGAGCTCATCTATTGAGCTGGGGACTTACTACCCCAGACAAAAAGCAT

CAGAAGGAACCTCCATTCCTTTGGATGGGATACGAACTCCATCCTGACAGATGGACAGTC

CAGCCGATAAAACTGCCAGAAAAAGACAGCTGGACTGTCAA--

>TJ20170316_11

-----GATCACTCTTTGGCAACGACCCCTTGTTGCCATAAGGATAGGGGGGCAAGTAAAG

GAAGCTCTACTAGATACAGGAGCAGATGATACAGTATTAGAAGACATAAATTTGCCAGGG

AAATGGAAACCAAAAATGATAGGAGGAATTGGAGGTTTTATCAAAGTAAGACAGTATGAA

CAGGTACCCATAGAAATCTGTGGACATAAAACTATAGGTACAGTGTTAATAGGACCTACA

CCTGTCAACATAATTGGGAGAAATCTGTTGACTCAGCTTGGTTGTACTTTAAATTTTCCA

ATCAGTCCTATTGACACTGTACCAGTAAAATTAAAGCCAGGAATGGATGGACCAAAGGTT

AAACAATGGCCATTAACAGAAGAAAAAATAAAAGCATTAATAGAAATTTGTAAAGAGATG

GAAGAGGAAGGAAAAATCTCAAAAATTGGGCCTGAAAATCCTTACAATACTCCAGTATTT

GCTATAAGGAAAAAGGACAGCACCAAATGGAGGAAATTAGTAGATTTCAGAGAGCTCAAT

AAAAGAACTCAGGACTTTTGGGAAGTTCAATTAGGAATACCGCATCCAGCAGGTTTAAAG

AAAAAGAAATCAGTAACAGTACTAGATGTAGGAGATGCATATTTCTCAGTTCCTTTAGAT

AAAAGCTTTAGAAAGTATACTGCATTTACCATACCTAGTACAAACAATGAGACACCAGGA

ATCAGATATCAGTACAATGTGCTGCCACAGGGATGGAAAGGATCACCGGCAATATTCCAG

AGTAGCATGACAAAGATCTTAGAACCTTTTAGAATAAAAAATCCAGAAATAGTCATCTAT

CAATATATGGATGACTTGTATGTAGGCTCCGATTTAGAAATAGGGCAGCACAGAATAAAA

ATAGAGGAGCTGAGAGCTCATCTATTGAGCTGGGGACTTACTACCCCAGACAAAAAGCAT

CAGAAGGAACCTCCATTCCTTTGGATAGGATACGAACTCCATCCTGACAGATGGACAGTC

CAGCCAGTAAAACTGCCAGAAAAAAGA----------------

>TJ20170315_1

------ATCACTCTTTGGCAACGACCCCTTGTCACAGTAAAAATAGGAGGACAGCTGAGA

GAAGCTCTACTAGATACAGGAGCAGATGATACAGTATTAGAAGAGATAAATTTGCCAGGA

AAATGGAAGCCAAAAATGATAGGGGGAATTGGAGGTTTTATCAAAGTAAGACAATATGAA

GAGATATGTATAGAAATTTGTGGAAAAAAGGCTATAGGTACAGTGTTAGTAGGACCTACA

CCTGTCAACATAATTGGACGAAATATGTTGACTCAGCTTGGTTGTACTCTAAATTTCCCA

ATTAGTCCTATTGACACTGTACCAGTAACATTAAAGCCAGGAATGGATGGACCAAAGGTT

AAACAGTGGCCATTGACAGAAGAAAAAATAAAAGCATTAACAGAAATTTGTAAGGAAATG

GAAAAGGAAGGAAAAATCTCAAAAATTGGGCCTGAAAATCCATATAATACTCCAGTGTTT

GCTATAAAGAAAAAGGACAGCACCAAATGGAGGAAATTAGTAGATTTCAGAGAGCTCAAT

AAAAGAACTCAGGACTTTTGGGAAGTTCAATTAGGAATACCGCATCCAGCAGGATTAAAA

AAGAAAAAATCAGTAACAGTACTAGATGTGGGAGATGCATATTTTTCAGTCCCTTTAGAT

AAAGACTTTAGAAAGTATACTGCATTCACCATACCTAGTATAAACAATGAAACACCAGGA

ATCAGATATCAGTACAATGTGCTGCCACAGGGATGGAAAGGATCTCCGGCAATATTCCAG

TGTAGCATGACAAAAATCTTAGAGCCCTTTAGAAGAAAAAATCCAGAGATGATTATCTAT

CAATATATGGATGACCTGTATGTAGGATCTGATTTAGAAATAGGGCAGCACAGAACAAAA

ATAGAGGAGCTAAGAGCTCATCTGTTGAGCTGGGGATTTACTACACCAGACAAAAAGCAT

CAGAAGGAACCACCATTTCTTTGGATGGGATATGAACTCCATCCGGACAGATGGACAGTC

CAGCCTATAGAACTACCAAAAAAA-------------------

>TJ20170315_2

------ATCACTCTTTGGCAACGACCCCTTGTTGCCATAAGGATAGGGGGGCAAGTAAAG

GAAGCTCTACTAGATACAGGAGCAGATGATACAGTATTAGAAGAAATAAATTTGCCAGGG

AAATGGAAACCAAAATTGATAGGGGGAATTGGAGGTTTTATCAAAGTAAGACAGTATGAA

CAGGTACCCATAGAAATCTGTGGACATAAAACTATAGGTACAGTATTAATAGGACCTACA

CCTGTCAACATAATTGGGAGAAATCTGTTGACTCAGCTTGGTTGTACTTTAAATTTTCCA

ATCAGTCCTATTGACACTGTACCAGTAAAATTAAAGCCAGGAATGGATGGACCAAAGGTT

AAACAATGGCCATTAACAGAAGAAAAAATAAAAGCATTAATAGAAATTTGTAAAGAGATG

GAAGAGGAAGGAAAAATCTCAAAAATTGGGCCTGAAAATCCTTACAATACTCCAGTATTT

GCTATAAGGAAAAAGGACAGCACCAAATGGAGGAAATTAGTAGATTTCAGAGAGCTCAAT

AAAAGAACTCAGGACTTTTGGGAAGTTCAATTAGGAATACCGCATCCAGCAGGTTTAAAG

AAAAAGAAATCAGTAACAGTACTAGATGTGGGAGATGCATATTTCTCAGTTCCTTTAGAT

AAAAGCTTTAGAAAGTATACTGCATTTACCATACCTAGTACAAACAATGAGACACCAGGA

ATCAGATATCAGTACAATGTGCTGCCACAGGGATGGAAAGGATCACCGGCAATATTCCAG

AGTAGCATGACAAAGATCTTAGAACCTTTTAGAATAAAAAATCCAGAAATAGTCATCTAT

CAATATATGGATGACTTGTATGTAGGCTCCGATTTAGAAATAGGGCAGCACAGAATAAAA

ATAGAGGAGCTGAGAGCTCATCTATTGAGCTGGGGACTTACTACCCCAGACAAAAAGCAT

CAGAAGGAACCTCCATTCCTTTGGATGGGATACGAACTCCATCCTGACAGATGGACAGTC

CAGCCAATAAAACTGCCAGAAAAAGACAGCTGGACTG------

>TJ20170315_3

-----GATCACTCTTTGGCAACGACCCCTTGTTGCCATAAGGATAGGGGGGCAAGTAAAG

GAAGCTCTACTAGATACAGGAGCAGATGATACAGTATTAGAAGAAATAAATTTGCCAGGG

AAATGGAAACCAAAATTGATAGGGGGAATTGGAGGTTTTATCAAAGTAAGACAGTATGAA

CAGGTACCCATAGAAATCTGTGGACATAAAACTATAGGTACAGTATTAATAGGACCTACA

CCTGTCAACATAATTGGGAGAAATCTGTTGACTCAGCTTGGTTGTACTTTAAATTTTCCA

ATCAGTCCTATTGACACTGTACCAGTAAAATTAAAGCCAGGAATGGATGGACCAAAGGTT

AAACAATGGCCATTAACAGAAGAAAAAATAAAAGCATTAATAGAAATTTGTAAAGAGATG

GAAGAGGAAGGAAAAATCTCAAAAATTGGGCCTGAAAATCCTTACAATACTCCAGTATTT

GCTATAAGGAAAAAGGACAGCACCAAATGGAGGAAATTAGTAGATTTCAGAGAGCTCAAT

AAAAGAACTCAGGACTTTTGGGAAGTTCAATTAGGAATACCGCATCCAGCAGGTTTAAAG

AAAAAGAAATCAGTAACAGTACTAGATGTGGGAGATGCATATTTCTCAGTTCCTTTAGAT

AAAAGCTTTAGAAAGTATACTGCATTTACCATACCTAGTACAAACAATGAGACACCAGGA

ATCAGATATCAGTACAATGTGCTGCCACAGGGATGGAAAGGATCACCGGCAATATTCCAG

AGTAGCATGACAAAGATCTTAGAACCTTTTAGAATAAAAAATCCAGAAATAGTCATCTAT

CAATATATGGATGACTTGTATGTAGGCTCCGATTTAGAAATAGGGCAGCACAGAATAAAA

ATAGAGGAGCTGAGAGCTCATCTATTGAGCTGGGGACTTACTACCCCAGACAAAAAGCAT

CAGAAGGAACCTCCATTCCTTTGGATGGGATACGAACTCCATCCTGACAGATGGACAGTC

CAGCCAATAAAACTGCCAGAAAAAGACAGCTGGACTGTC----

>TJ20170315_4

CCTCAGATCACTCTTTGGCAACGACCCCTTGTTGCCATAAGGATAGGGGGGCAAGTAAAG

GAAGCTCTACTAGATACAGGAGCAGATGATACAGTATTAGAAGACATAAATTTGCCAGGG

AAATGGAAACCAAAAATGATAGGGGGAATTGGAGGTTTTATCAAAGTAAGACAGTATGAA

CAGGTACCCATAGAAATCTGTGGACATAAAACTATAGGTACAGTATTAATAGGACCTACA

CCTGTCAACATAATTGGGAGAAATCTGTTGACTCAGCTTGGTTGTACTTTAAATTTTCCA

ATCAGTCCTATTGACACTGTACCAGTAAAATTAAAGCCAGGAATGGATGGACCAAAGGTT

AAACAATGGCCATTAACAGAAGAAAAAATAAAAGCATTAATAGAAATTTGTAAAGAGATG

GAAGAGGAAGGAAAAATCTCAAAAATTGGGCCTGAAAATCCTTACAATACTCCAGTATTT

GCTATAAGGAAAAAGGACAGCACCAAATGGAGGAAATTAGTAGATTTCAGAGAGCTCAAT

AAAAGAACTCAGGACTTTTGGGAAGTTCAATTAGGAATACCGCATCCAGCAGGTTTAAAG

AAAAAGAAATCAGTAACAGTACTAGATGTGGGAGATGCATATTTCTCAGTTCCTTTAGAT

AAAAGCTTTAGAAAGTATACTGCATTTACCATACCTAGTACAAACAATGAGACACCAGGA

ATCAGATATCAGTACAATGTGCTGCCACAGGGATGGAAAGGATCACCGGCAATATTCCAG

AGTAGCATGACAAAGATCTTAGAACCTTTTAGAATAAAAAATCCAGAAATAGTCATCTAT

CAATATATGGATGACTTGTATGTAGGCTCCGATTTAGAAATAGGGCAGCACAGAATAAAA

ATAGAGGAGCTGAGAGCTCATCTATTGAGCTGGGGACTTACTACCCCAGACAAAAAGCAT

CAGAAGGAACCTCCATTCCTTTGGATGGGATACGAACTCCATCCTGACAGATGGACAGTC

CAGCCAATAAAACTGCCAGAAAAAGACAGCTGGACTGTC----

>TJ20170315_5

-----GATCACTCTTTGGCAACGACCCCTTGTTGCCATAAGGATAGGGGGGCAAGTAAAG

GAAGCTCTACTAGATACAGGAGCAGATGATACAGTATTAGAAGAAATAAATTTGCCAGGG

AAATGGAAACCAAAATTGATAGGGGGAATTGGAGGTTTTATCAAAGTAAGACAGTATGAA

CAGGTACCCATAGAAATCTGTGGACATAAAACTATAGGTACAGTATTAATAGGACCTACA

CCTGTCAACATAATTGGGAGAAATCTGTTGACTCAGCTTGGTTGTACTTTAAATTTTCCA

ATCAGTCCTATTGACACTGTACCAGTAAAATTAAAGCCAGGAATGGATGGACCAAAGGTT

AAACAATGGCCATTAACAGAAGAAAAAATAAAAGCATTAATAGAAATTTGTAAAGAGATG

GAAGAGGAAGGAAAAATCTCAAAAATTGGGCCTGAAAATCCTTACAATACTCCAGTATTT

GCTATAAGGAAAAAGGACAGCACCAAATGGAGGAAATTAGTAGATTTCAGAGAGCTCAAT

AAAAGAACTCAGGACTTTTGGGAAGTTCAATTAGGAATACCGCATCCAGCAGGTTTAAAG

AAAAAGAAATCAGTAACAGTACTAGATGTGGGAGATGCATATTTCTCAGTTCCTTTAGAT

AAAAGCTTTAGAAAGTATACTGCATTTACCATACCTAGTACAAACAATGAGACACCAGGA

ATCAGATATCAGTACAATGTGCTGCCACAGGGATGGAAAGGATCACCGGCAATATTCCAG

AGTAGCATGACAAAGATCTTAGAACCTTTTAGAATAAAAAATCCAGAAATAGTCATCTAT

CAATATATGGATGACTTGTATGTAGGCTCCGATTTAGAAATAGGGCAGCACAGAATAAAA

ATAGAGGAGCTGAGAGCTCATCTATTGAGCTGGGGACTTACTACCCCAGACAAAAAGCAT

CAGAAGGAACCTCCATTCCTTTGGATGGGATACGAACTCCATCCTGACAGATGGACAGTC

CAGCCAATAAAACTGCCAGAAAAAGACAGCTGGACTG------

>TJ20170315_6

-----GATCACTCTTTGGCAACGACCCCTTGTTGCCATAAGGATAGGGGGGCAAGTAAAG

GAAGCTCTACTAGATACAGGAGCAGATGATACAGTATTAGAAGAAATAAATTTGCCAGGG

AAATGGAAACCAAAATTGATAGGGGGAATTGGAGGTTTTATCAAAGTAAGACAGTATGAA

CAGGTACCCATAGAAATCTGTGGACATAAAACTATAGGTACAGTATTAATAGGACCTACA

CCTGTCAACATAATTGGGAGAAATCTGTTGACTCAGCTTGGTTGTACTTTAAATTTTCCA

ATCAGTCCTATTGACACTGTACCAGTAAAATTAAAGCCAGGAATGGATGGACCAAAGGTT

AAACAATGGCCATTAACAGAAGAAAAAATAAAAGCATTAATAGAAATTTGTAAAGAGATG

GAAGAGGAAGGAAAAATCTCAAAAATTGGGCCTGAAAATCCTTACAATACTCCAGTATTT

GCTATAAGGAAAAAGGACAGCACCAAATGGAGGAAATTAGTAGATTTCAGAGAGCTCAAT

AAAAGAACTCAGGACTTTTGGGAAGTTCAATTAGGAATACCGCATCCAGCAGGTTTAAAG

AAAAAGAAATCAGTAACAGTACTAGATGTGGGAGATGCATATTTCTCAGTTCCTTTAGAT

AAAAGCTTTAGAAAGTATACTGCATTTACCATACCTAGTACAAACAATGAGACACCAGGA

ATCAGATATCAGTACAATGTGCTGCCACAGGGATGGAAAGGATCACCGGCAATATTCCAG

AGTAGCATGACAAAGATCTTAGAACCTTTTAGAATAAAAAATCCAGAAATAGTCATCTAT

CAATATATGGATGACTTGTATGTAGGCTCCGATTTAGAAATAGGGCAGCACAGAATAAAA

ATAGAGGAGCTGAGAGCTCATCTATTGAGCTGGGGACTTACTACCCCAGACAAAAAGCAT

CAGAAGGAACCTCCATTCCTTTGGATGGGATACGAACTCCATCCTGACAGATGGACAGTC

CAGCCAATA----------------------------------

>TJ20170315_7

----AGATCACTCTTTGGCAGCGACCCCTTGTTGCCATAAGGATAGGGGGGCAAGTAAAG

GAAGCTCTACTAGATACAGGAGCAGATGATACAGTATTAGAAGAAATGGATTTGCCAGGG

AAATGGAAACCAAAAATGATAGGGGGAATTGGAGGTTTTATCAAAGTAAGACAGTATGAA

CAGGTACCCATAGAAATCTGTGGACATAAAACTATAGGTACAGTATTAATAGGACCTACA

CCTGTCAACATAATTGGGAGAAATCTGTTGACTCAGCTTGGTTGTACTTTAAATTTTCCA

ATCAGTCCTATTGACACTGTACCAGTAAAATTAAAGCCAGGAATGGATGGACCAAAGGTT

AAACAATGGCCATTAACAGAAGAAAAAATAAAAGCATTAATAGAAATTTGTAAAGAGATG

GAAGAGGAAGGAAAAATCTCAAAAATTGGGCCTGAAAATCCTTACAATACTCCAGTATTT

GCTATAAGGAAAAAGGACAGCACCAAATGGAGGAAATTAGTAGATTTCAGAGAGCTCAAT

AAAAGAACTCAGGACTTTTGGGAAGTTCAATTAGGAATACCGCATCCAGCAGGTTTAAAG

AAAAAGAAATCAGTAACAGTACTAGATGTGGGAGATGCATATTTCTCAGTTCCTTTAGAT

AAAAGCTTTAGAAAGTATACTGCATTTACCATACCTAGTATAAACAATGAGACACCAGGA

ATCAGATATCAGTACAATGTGCTGCCACAGGGATGGAAAGGATCACCGGCAATATTCCAG

AGTAGCATGACAAAGATCTTAGAACCTTTTAGAATAAAAAATCCAGAAATAGTCATCTAT

CAATATATGGATGACTTGTATGTAGGCTCCGATTTAGAAATAGGGCAGCACAGAATAAAA

ATAGAGGAGCTGAGAGCTCATCTATTGAGCTGGGGACTTACGACCCCAGACAAAAAGCAT

CAGAAGGAACCTCCATTCCTTTGGATGGGATACGAACTCCATCCTGACAGATGGACAGTC

CAGCCAATAAAACTGCCAGAAAAAGACAGCTGGACTG------

>TJ20170315_8

-----GATCACTCTTTGGCAACGACCCCTTGTTGCCATAAGGATAGGGGGGCAAGTAAAG

GAAGCTCTACTAGATACAGGAGCAGATGATACAGTATTAGAAGAAATGAATTTGCCAGGG

AAATGGAAACCAAAATTGATAGGGGGAATTGGAGGTTTTATCAAAGTAAGACAGTATGAA

CAGGTACCCATAGAAATCTGTGGACATAAAACTATAGGTACAGTATTAATAGGACCTACA

CCTGTCAACATAATTGGGAGAAATCTGTTGACTCAGCTTGGTTGTACTTTAAATTTTCCA

ATCAGTCCTATTGACACTGTACCAGTAAAATTAAAGCCAGGAATGGATGGACCAAAGGTT

AAACAATGGCCATTAACAGAAGAAAAAATAAAAGCATTAATAGAAATTTGTAAAGAGATG

GAAGAGGAAGGAAAAATCTCAAAAATTGGGCCTGAAAATCCTTACAATACTCCAGTATTT

GCTATAAGGAAAAAGGACAGCACCAAATGGAGGAAATTAGTAGATTTCAGAGAGCTCAAT

AAAAGAACTCAGGACTTTTGGGAAGTTCAATTAGGAATACCGCATCCAGCAGGTTTAAAG

AAAAAGAAATCAGTAACAGTACTAGATGTGGGAGATGCATATTTCTCAGTTCCTTTAGAT

AAAAGCTTTAGAAAGTATACTGCATTTACCATACCTAGTACAAACAATGAGACACCAGGA

ATCAGATATCAGTACAATGTGCTGCCACAGGGATGGAAAGGATCACCGGCAATATTCCAG

AGTAGCATGACAAAGATCTTAGAACCTTTTAGAATAAAAAATCCAGAAATAGTCATCTAT

CAATATATGGATGACTTGTATGTAGGCTCCGATTTAGAAATAGGGCAGCACAGAATAAAA

ATAGAGGAGCTGAGAGCTCATCTATTGAGCTGGGGACTTACTACCCCAGACAAAAAGCAT

CAGAAGGAACCTCCATTCCTTTGGATGGGATACGAACTCCATCCTGACAGATGGACAGTC

CAGCCAATAAAACTGCCAGAAAAAGACAGCTGGACTGTC----

>TJ20170315_9

-------TCACTCTTTGGCAACGACCCCTTGTCACAGTAAAAATAGGAGGACAGCTGAGA

GAAGCTCTACTAGATACAGGAGCAGATGATACAGTATTAGAAGAGATAAATTTGCCAGGA

AAATGGAAGCCAAAATTGATAGGGGGAATTGGAGGTTTTATCAAAGTAAGACAATATGAA

GAGATATGTATAGAAATTTGTGGAAAAAAGGCTATAGGTACAGTATTAGTAGGACCTACA

CCTGTCAACATAATTGGACGAAATATGTTGACTCAGCTTGGTTGTACTTTAAATTTCCCA

ATTAGTCCTATTGACACTGTACCAGTAACATTAAAGCCAGGAATGGATGGACCAAAGGTT

AAACAGTGGCCATTGACAGAAGAAAAAATAAAAGCATTAACAGAAATTTGTAAGGAAATG

GAAGAGGAAGGAAAAATCTCAAAAATTGGGCCTGAAAATCCATATAATACTCCAGTGTTT

GCTATAAAGAAAAAGGACAGCACCAAATGGAGGAAATTAGTAGATTTCAGAGAGCTCAAT

AAAAGAACTCAGGACTTTTGGGAAGTTCAATTAGGAATACCGCATCCAGCAGGATTAAAA

AAGAAAAAATCAGTGACAGTACTAGATGTGGGAGATGCATATTTTTCAGTCCCTTTAGAT

AAAGACTTTAGGAAGTATACTGCATTCACCATACCTAGTATAAACAATGAGACACCAGGA

ATCAGATATCAGTACAATGTGCTGCCACAGGGATGGAAAGGATCTCCGGCAATATTCCAG

TGTAGCATGACAAAAATCTTAGAGCCCTTTAGAAGAAAAAATCCAGAGATGATTATCTAT

CAATATATGGATGATTTGTATGTAGGATCTGATTTAGAAATAGGGCAGCACAGAACAAAA

ATAGAGGAGCTAAGAGCTCATCTGTTGAGCTGGGGATTTACTACACCAGACAAAAAGCAT

CAGAAGGAACCACCATTTCTTTGGATGGGATATGAACTCCATCCGGACAGATGGACAGTC

CAGCCTATAGAACTACCAGAAAAA-------------------

>TJ20170315_10

----AGATCACTCTTTGGCAACGACCCCTTGTTGCCATAAGGATAGGGGGGCAAGTAAAG

GAAGCTCTACTAGATACAGGAGCAGATGATACAGTATTAGAAGAAACAAATTTGCCAGGG

AAATGGAAACCAAAATTGATAGGGGGAATTGGAGGTTTTATCAAAGTAAGACAGTATGAA

CAGGTACCCATAGAAATCTGTGGACATAAAACTATAGGTACAGTATTAATAGGACCTACA

CCTGTCAACATAATTGGGAGAAATCTGTTGACTCAGCTTGGTTGTACTTTAAATTTTCCA

ATCAGTCCTATTGACACTGTACCAGTAAAATTAAAGCCAGGAATGGATGGACCAAAGGTT

AAACAATGGCCATTAACAGAAGAAAAAATAAAAGCATTAATAGAAATTTGTAAAGAGATG

GAAGAGGAAGGAAAAATCTCAAAAATTGGGCCTGAAAATCCTTACAATACTCCAGTATTT

GCTATAAGGAAAAAGGACAGCACCAAATGGAGGAAATTAGTAGATTTCAGAGAACTCAAT

AAAAGAACTCAGGACTTTTGGGAAGTTCAATTAGGAATACCGCATCCAGCAGGTTTAAAG

AAAAAGAAATCAGTAACAGTACTAGATGTGGGAGATGCATATTTCTCAGTTCCTTTAGAT

AAAAGCTTTAGAAAGTATACTGCATTTACCATACCTAGTATAAACAATGAGACACCAGGA

ATCAGATATCAGTACAATGTGCTGCCACAGGGATGGAAAGGATCACCGGCAATATTCCAG

AGTAGCATGACAAAGATCTTAGAACCTTTTAGAATAAAAAATCCAGAAATAGTCATCTAT

CAATATATGGATGACTTGTATGTAGGCTCCGATTTAGAAATAGGGCAGCACAGAATAAAA

ATAGAGGAGCTGAGAGCTCATCTATTGAGCTGGGGACTTACTACCCCAGACAAAAAGCAT

CAGAAGGAACCTCCATTCCTTTGGATGGGATACGAACTCCATCCTGACAGATGGACAGTC

CAGCCAATAAAACTGCCAGAAAAAGACAGCTGGACTGTC----

>TJ20170315_11

----AGATCACTCTTTGGCAACGACCCCTTGTTGCCATAAGGATAGGGGGGCAAGTAAAG

GAAGCTCTACTAGATACAGGAGCAGATGATACAGTATTAGAAGAAATAAATTTGCCAGGG

AAATGGAAACCAAAATTGATAGGGGGAATTGGAGGTTTTATCAAAGTAAGACAGTATGAA

CAGGTACCCATAGAAATCTGTGGACATAAAACTATAGGTACAGTATTAATAGGACCTACA

CCTGTCAACATAATTGGGAGAAATCTGTTGACTCAGCTTGGTTGTACTTTAAATTTTCCA

ATCAGTCCTATTGACACTGTACCAGTAAAATTAAAGCCAGGAATGGATGGACCAAAGGTT

AAACAATGGCCATTAACAGAAGAAAAAATAAAAGCATTAATAGAAATTTGTAAAGAGATG

GAAGAGGAAGGAAAAATCTCAAAAATTGGGCCTGAAAATCCTTACAATACTCCAGTATTT

GCTATAAGGAAAAAGGACAGCACCAAATGGAGGAAATTAGTAGATTTCAGAGAGCTCAAT

AAAAGAACTCAGGACTTTTGGGAAGTTCAATTAGGAATACCGCATCCAGCAGGTTTAAAG

AAAAAGAAATCAGTAACAGTACTAGATGTGGGAGATGCATATTTCTCAGTTCCTTTAGAT

AAAAGCTTTAGAAAGTATACTGCATTTACCATACCTAGTACAAACAATGAGACACCAGGA

ATCAGATATCAGTACAATGTGCTGCCACAGGGATGGAAAGGATCACCGGCAATATTCCAG

AGTAGCATGACAAAGATCCTAGAACCTTTTAGAATAAAAAATCCAGAAATAGTCATCTAT

CAATATATGGATGACTTGTATGTAGGCTCCGATTTAGAAATAGGGCAGCACAGAATAAAA

ATAGAGGAGCTGAGAGCTCATCTATTGAGCTGGGGACTTACTACCCCAGACAAAAAGCAT

CAGAAGGAACCTCCATTCCTTTGGATGGGATACGAACTCCATCCTGACAGATGGACAGTC

CAGCCAATAAAACTGCCAGAAAAAGACAGCTGGACTGTC----

>TJ20170315_12

------ATCACTCTTTGGCAACGACCCCTTGTCACAGTAAAAATAGGAGGACAGCTGAGA

GAAGCTCTATTAGATACAGGAGCAGATGATACAGTATTAGAAGAGATAAATTTGCCAGGA

AAATGGAAGCCAAAAATGATAGGGGGGATTGGAGGTTTTATCAAAGTAAGACAATATGAA

GAGATATGTATAGAAATTTGTGGAAAAAAGGCTATAGGTACAGTGTTAGTAGGACCTACA

CCTGTCAACATAATTGGCCGAAATATGTTGACTCAGCTTGGTTGTACTTTAAATTTCCCA

ATTAGTCCTATTGACACTGTACCAGTAACATTAAAGCCAGGAATGGATGGACCAAAGGTT

AAACAGTGGCCATTGACAGAAGAAAAAATAAAAGCATTAACAGAAATTTGTAAGGAAATG

GAAGAGGAAGGAAAAATCTCAAAAATTGGGCCTGAAAATCCATATAATACTCCAGTGTTT

GCTATAAAGAAAAAGGACAGCACCAAATGGAGGAAATTAGTAGATTTCAGAGAGCTCAAT

AAAAGAACTCAGGACTTTTGGGAAGTTCAATTAGGAATACCGCATCCAGCAGGATTAAAA

AAGAAAAAATCAGTGACAGTACTAGATGTGGGAGATGCATATTTTTCAGTCCCTTTAGAT

AAAACTTTTAGAAAGTATACTGCATTCACCATACCTAGTATAAACAATGAAACACCAGGA

ATCAGATATCAGTACAATGTGCTGCCACAGGGATGGAAAGGATCTCCGGCAATATTCCAG

TGTAGCATGACAAAAATCTTAGAGCCCTTTAGAAGAAAAAATCCAGAGATGATTATCTAT

CAATATATGGATGATTTGTATGTAGGATCTGACTTAGAAATAGGGCAGCACAGAACAAAA

ATAGAGGAGCTAAGAGCTCATCTGTTGAGCTGGGGATTTACTACACCAGACAAAAAGCAT

CAGAAGGAACCACCATTTCTTTGGATGGGATATGAACTCCATCCGGACAGATGGACAGTC

CA-----------------------------------------

>TJ20170315_13

----AGATCACTCTTTGGCAACGACCCCTTGTTGCCATAAGGATAGGGGGGCAAGTAAAG

GAAGCTCTACTAGATACAGGAGCAGATGATACAGTATTAGAAGAAATAAGTTTGCCAGGG

AAATGGAAACCAAAATTGATAGGGGGAATTGGAGGTTTTATCAAAGTAAGACAGTATGAA

CAGGTACCCATAGAAATCTGTGGACATAAAACTATAGGTACAGTATTAATAGGACCTACA

CCTGTCAACATAATTGGGAGAAATCTGTTGACTCAGCTTGGTTGTACTTTAAATTTTCCA

ATCAGTCCTATTGACACTGTACCAGTAAAATTAAAGCCAGGAATGGATGGACCAAAGGTT

AAACAATGGCCATTAACAGAAGAAAAAATAAAAGCATTAATAGAAATTTGTAAAGAGATG

GAAGAGGAAGGAAAAATCTCAAAAATTGGGCCTGAAAATCCTTACAATACTCCAGTATTT

GCTATAAGGAAAAAGGACAGCACCAAATGGAGGAAATTAGTAGATTTCAGAGAGCTCAAT

AAAAGAACTCAGGACTTTTGGGAAGTTCAATTAGGAATACCGCATCCAGCAGGTTTAAAG

AAAAAGAAATCAGTAACAGTACTAGATGTGGGAGATGCATATTTCTCAGTTCCTTTAGAT

AAAAGCTTTAGAAAGTATACTGCATTTACCATACCTAGTACAAACAATGAGACACCAGGA

ATCAGATATCAGTACAATGTGCTGCCACAGGGATGGAAAGGATCACCGGCAATATTCCAG

AGTAGCATGACAAAGATCTTAGAACCTTTTAGAATAAAAAATCCAGAAATAGTCATCTAT

CAATATATGGATGACTTGTATGTAGGCTCCGATTTAGAAATAGGGCAGCACAGAATAAAA

ATAGAGGAGCTGAGAGCTCATCTATTGAGCTGGGGACTTACTACCCCAGACAAAAAGCAT

CAGAAGGAACCTCCATTCCTTTGGATGGGATACGAACTCCATCCTGACAGATGGACAGTC

CAGCCAATAAAACTGCCAGAAAAAGACAGCTGGACTGT-----

>TJ20170315_14

------ATCACTCTTTGGCAACGACCCCTTGTTGCCATAAGGATAGGGGGGCAAGTAAAG

GAAGCTCTACTAGATACAGGAGCAGATGATACAGTATTAGAAGAAATAAATTTGCCAGGG

AAATGGAAACCAAAATTGATAGGGGGAATTGGAGGTTTTATCAAAGTAAGACAGTATGAA

CAGGTACCCATAGAAATCTGTGGACATAAAACTATAGGTACAGTATTAATAGGACCTACA

CCTGTCAACATAATTGGGAGAAATCTGTTGACTCAGCTTGGTTGTACTTTAAATTTTCCA

ATCAGTCCTATTGACACTGTACCAGTAAAATTAAAGCCAGGAATGGATGGACCAAAGGTT

AAACAATGGCCATTAACAGAAGAAAAAATAAAAGCATTAATAGAAATTTGTAAAGAGATG

GAAGAGGAAGGAAAAATCTCAAAAATTGGGCCTGAAAATCCTTACAATACTCCAGTATTT

GCTATAAGGAAAAAGGACAGCACCAAATGGAGGAAATTAGTAGATTTCAGAGAGCTCAAT

AAAAGAACTCAGGACTTTTGGGAAGTTCAATTAGGAATACCGCATCCAGCAGGTTTAAAG

AAAAAGAAATCAGTAACAGTACTAGATGTGGGAGATGCATATTTCTCAGTTCCTTTAGAT

AAAAGCTTTAGAAAGTATACTGCATTTACCATACCTAGTACAAACAATGAGACACCAGGA

ATCAGATATCAGTACAATGTGCTGCCACAGGGATGGAAAGGATCACCGGCAATATTCCAG

AGTAGCATGACAAAGATCTTAGAACCTTTTAGAATAAAAAATCCAGAAATAGTCATCTAT

CAATATATGGATGACTTGTATGTAGGCTCCGATTTAGAAATAGGGCAGCACAGAATAAAA

ATAGAGGAGCTGAGAGCTCATCTATTGAGCTGGGGACTTACTACCCCAGACAAAAAGCAT

CAGAAGGAACCTCCATTCCTTTGGATGGGATACGAACTCCATCCTGACAGATGGACAGTC

CAGCCAATAAAACTGCCAGAAAAAGACAGCTGGACTGTC----

1. *env* SGA sequences of the couple

>TJ20170317_1

AGAAGAAGAGATAATAATCAGATCTGAAAATCTCACAAACAATGCCAAAACCATAATAGT

GCACCTTAATGAATCTGTAGAAATCAGTTGTACCAGACCCTCCAATAATACAATAACAAG

CATAAATATAGGACCAGGACGAGCATTCTATAGAACAGGAGACATAGTAGGAGATATAAG

ACAAGCCTTTTGTGAAATTAATGGAGCAAAATGGAACAAAACTTTAGGACAGGTAGCTAA

AAAATTAAAAGAGCACTTTAATAAGACAATAGAATTCCAGCCGCCCTCAGGGGGAGATCT

AGAAATTACAATGCATCATTTTAATTGTAGAGGGGAATTTTTCTATTGCAATACAACAAA

GCTGTTCAATAGTACTTGGATAGGAAATGAGACCATAGGGGAAGGTAATGACACTATCAT

ACTTCCATGCAGGATAAAGCAAATTATAAACATGTGGCAAGGAGTAGGACAAGCAATGTA

TGCCCCCCCCATCAGAGGAATAATTAAATGTGCATCAAATATTACAGGAATACTATTGAC

AAGAGATGGTGGTAGAAATGCTAGCGAGCCTGAGACTTTTAGACCTGGAGGAGGAA

>TJ20170317_2

AGAAGAAGAGATAATAATCAGATCTGAAAATCTCACAAACAATGCCAAAACCATAATAGT

GCACCTTAATGAATCTGTAGAAATCAGTTGTACCAGACCCTCCAATAATACAATAACAAG

CATAAATATAGGACCAGGACGAGCATTCTATAGAACAGGAGACATAGTAGGAGATATAAG

ACAAGCCTTTTGTGAAATTAATGGAGCAAAATGGAACAAAACTTTAGGACAGGTAGCTAA

AAAATTAAAAGAGCACTTTAATAAGACAATAGAATTCCAGCCGCCCTCAGGGGGAGATCT

AGAAATTACAATGCATCATTTTAATTGTAGAGGGGAATTTTTCTATTGCAATACAACAAA

GCTGTTCAATAGTACTTGGATAGGAAATGAGACCATAGGGGAAGATAATGACACTATCAT

ACTTCCATGCAGGATAAAGCAAATTATAAACATGTGGCAAGGAGTAGGACAAGCAATGTA

TGCTCCCCCCATCAGAGGAATAATTAAATGTGCATCAAATATTACAGGAATACTATTGAC

AAGAGATGGTGGTAGAAATGCTAGCGAGCCTGAGACTTTTAGACCTGGAGGAGGAA

>TJ20170317_3

AGAAGAAGAGATAATAATCAGATCTGAAAATCTCACAAACAATGCCAAAACCATAATAGT

GCACCTTAATGAATCTGTAGAAATCAGTTGTACCAGACCCTCCAATAATACAATAACAAG

CATAAATATAGGACCAGGACGAGCATTCTATAGAACAGGAGACATAGTAGGAGATATAAG

ACAAGCCTTTTGTGAAATTAATGGAGCAAAATGGAACAAAACTTTAGGACAGGTAGCTAA

AAAATTAAAAGAGCACTTTAATAAGACAATAGAATTCCAGCCGCCCTCAGGGGGAGATCT

AGAAATTACAATGCATCATTTTAATTGTAGAGGGGAATTTTTCTATTGCAATACAACAAA

GCTGTTCAATAGTACTTGGATAGGAAATGAGACCATAGAGGAAGGTAATGACACTATCAT

ACTTCCATGCAGGATAAAGCAAATTATAAACATGTGGCAAGGAGTAGGACAAGCAATGTA

TGCCCCCCCCATCAGAGGAATAATTAAATGTGCATCAAATATTACAGGAATACTATTGAC

AAGAGATGGTGGTAGAAATGCTAGCGAGCCTGAGACTTTTAGACCTGGAGGAGGAA

>TJ20170317_4

AGAAGAAGAGATAATAATCAGATCTGAAAATCTCACAAACAATGCCAAAACCATAATAGT

GCACCTTAATGAATCTGTAGAAATCAGTTGTACCAGACCCTCCAATAATACAATAACAAG

CATAAATATAGGACCAGGACGAGCATTCTATAGAACAGGAGACATAGTAGGAGATATAAG

ACAAGCCTTTTGTGAAATTAATGGAGCAAAATGGAACAAAACTTTAGGACAGGTAGCTAA

AAAATTAAAAGAGCACTTTAATAAGACAATAGAATTCCAGCCGCCCTCAGGGGGAGATCT

AGAAATTACAATGCATCATTTTAATTGTAGAGGGGAATTTTTCTATTGCAATACAACAAA

GCTGTTCAATAGTACTTGGATAGGAAATGAGACCATAGGGGAAGGTAATGACACTATCAT

ACTTCCATGCAGGATAAAGCAAATTATAAACATGTGGCAAGGAGTAGGACAAGCAATGTA

TGCCCCCCCCATCAGAGGAATAATTAAATGTGCATCAAATATTACAGGAATACTATTGAC

AAGAGATGGTGGTAGAAATGCTAGCGAGCTTGAGACTTTTAGACCTGGAGGAGGAA

>TJ20170317_5

AGAAGAAGAGATAATAATCAGATCTGAAGATCTCACAAACAATGCCAAAACCATAATAGT

GCACCTTAATGAATCTGTAGAAATCAGTTGTACCAGACCCTCCAATAATACAATAACAAG

CATAAATATAGGACCAGGACGAGCATTCTATAGAACAGGAGACATAGTAGGAGATATAAG

ACAAGCCTTTTGTGAAATTAATGGAGCAAAATGGAACAAAACTTTAGGACAGGTAGCTAA

AAAATTAAAAGAGCACTTTAATAAGACAATAGAATTCCAGCCGCCCTCAGGGGGAGATCT

AGAAATTACAATGCATCATTTTAATTGTAGAGGGGAATTTTTCTATTGCAATACAACAAA

GCTGTTCAATAGTACTTGGATAGGAAATGAGACCATAAGGGAAGGTAATGACACTATCAT

ACTTCCATGCAGGATAAAGCAAATTATAAACATGTGGCAAGGAGTAGGACAAGCAATGTA

TGCTCCCCCCATCAGAGGAATAATTAAATGTGCATCAAATATTACAGGAATACTATTGAC

AAGAGATGGTGGTAGAAATACTAGCGAGCTTGAGACTTTTAGACCTGGAGGAGGAA

>TJ20170317_6

AGAAGAAGAGATAATAATCAGATCTGAAAATCTCACAAACAATGCCAAAACCATAATAGT

GCACCTTAATGAATCTGTAGAAATCAGTTGTACCAGACCCTCCAATAATACAATAACAAG

CATAAATATAGGACCAGGACGAGCATTCTATAGAACAGGAGACATAGTAGGAGATATAAG

ACAAGCCTTTTGTGAAATTAATGGAGCAAAATGGAACAAAACTTTAGGACAGGTAGCTAA

AAAATTAAAAGAGCACTTTAATAAGACAATAGAATTCCAGCCGCCCTCAGGGGGAGATCT

AGAAATTACAATGCATCATTTTAATTGTAGAGGGGAATTTTTCTATTGCAATACAACAAA

GCTGTTCAATAGTACTTGGATAGGAAATGAGACCATAAGGGAAGGTAATGACACTATCAT

ACTTCCATGCAGGATAAAGCAAATTATAAACATGTGGCAAGGAGTAGGACAAGCAATGTA

TGCCCCCCCCATCAGAGGAATAATTAAATGTGCATCAAATATTACAGGAATACTATTGAC

AAGAGATGGTGGTAGAAATGCTAGCGAGCCTGAGACTTTTAGACCTGGAGGAGGAA

>TJ20170317_7

AGAAGAAGAGATAATAATCAGATCTGAAAATCTCACAAACAATGCCAAAACCATAATAGT

GCACCTTAATGAATCTGTAGAAATCAGTTGTACCAGACCCTCCAATAATACAATAACAAG

CATAAATATAGGACCAGGACGAGCATTCTATAGAACAGGAGACATAGTAGGAGATATAAG

ACAAGCCTTTTGTGAAATTAATGGAGCAAAATGGAACAAAACTTTAGGACAGGTAGCTAA

AAAATTAAAAGAGCACTTTAATAAGACAATAGAATTCCAGCCGCCCTCAGGGGGAGATCT

AGAAATTACAATGCATCATTTTAATTGTAGAGGGGAATTTTTCTATTGCAATACAACAAA

GCTGTTCAATAGTACTTGGATAGGAAATGAGACCATAGAGGAAGGTAATGACACTATCAT

ACTTCCATGCAGGATAAAACAAATTATAAACATGTGGCAAGGAGTAGGACAAGCAATGTA

TGCCCCCCCCATCAGAGGAATAATTAAATGTGCATCAAATATTACAGGAATACTATTGAC

AAGAGATGGTGGTAGAAATGCTAGCGAGCCTGAGACTTTTAGACCTGGAGGAGGAA

>TJ20170317_8

AGAAGAAGAGATAATAATCAGATCTGAAAATCTCACAAACAATGCCAAAACCATAATAGT

GCACCTTAATGAATCTGTAGAAATCAGTTGTACCAGACCCTCCAATAATACAATAACAAG

CATAAATATAGGACCAGGACGAGCATTCTATAGAACAGGAGACATAGTAGGAGATATAAG

ACAAGCCTTTTGTGAAATTAATGGAGCAAAATGGAACAAAACTTTAGGACAGGTAGCTAA

AAAATTAAAAGAGCACTTTAATAAGACAATAGAATTCCAGCCGCCCTCAGGGGGAGATCT

AGAAATTACAATGCATCATTTTAATTGTAGAGGGGAATTTTTCTATTGCAATACAACAAA

GCTGTTCAATAGTACTTGGATAGGAAATGAGACCATAGGGGAAGGTAATGACACTATCAT

ACTTCCATGCAGGATAAAGCAAATTATAAACATGTGGCAAGGAGTAGGACAAGCAATGTA

TGCCCCCCCCATCAGAGGAATAATTAAATGTGCATCAAATATTACAGGAATACTATTGAC

AAGAGATGGTGGTAGAAATGCTAGCGAGCTTGAGACTTTTAGACCTGGAGGAGGAA

>TJ20170317_9

AGAAGAAGAGATAATAATCAGATCTGAAAATCTCACAAACAATGCAAAAACCATAATAGT

GCACCTTAATGAATCTGTAGAAATCAGTTGTACCAGACCCTCCAATAATACAATAACAAG

CATAAATATAGGACCAGGACGAGCATTCTATAGAACAGGAGACATAGTAGGAGATATAAG

ACGAGCCTTTTGTGAAATTAATGGAGCAAAATGGAACAAAACTTTAGGACAGGTAGCTAA

AAAATTAAAAGAGCACTTTAATAAGACAATAGAATTCCAGCCGCCCTCAGGGGGAGATCT

AGAAATTACAATGCATCATTTTAATTGTAGAGGGGAATTTTTCTATTGCAATACAACAAA

GCTGTTCAATAGTACCTGGATAGGAAATGAGACCATAGGGGGAGGTAATGACACTATCAT

ACTTCCATGCAGGATAAAGCAAATTATAAACATGTGGCAAGGAGTAGGACAAGCAATGTA

TGCCCCCCCCATCAGAGGAATAATTAAATGTGCATCAAATATTACAGGAATACTATTGAC

AAGAGATGGTGGTAGAAATGCTAGCGAGCCTGAGACTTTTAGACCTGGAGGAGGAA

>TJ20170317_10

AGAAGAAGAGATAATAATCAGATCTGAAAATCTCACAAACAATGCCAAAACCATAATAGT

GCACCTTAATGAATCTGTAGAAATCAGTTGTACCAGACCCTCCAATAATACAATAACAAG

CATAAATATAGGACCAGGACGAGCATTCTATAGAACAGGAGACATAGTAGGAGATATAAG

ACAAGCCTTTTGTGAAATTAATGGAGCAAAATGGAACAAAACTTTAGGACAGGTAGCTAA

AAAATTAAAAGAGCACTTTAATAAGACAATAGAATTCCAGCCGCCCTCAGGGGGAGATCT

AGAAATTACAATGCATCATTTTAATTGTAGAGGGGAATTTTTCTATTGCAATACAACAAA

GCTGTTCAATAGTACTTGGATAGGAAATGAGACCATAGAGGAAGGTAATGACACTATCAT

ACTTCCATGCAGGATAAAGCAAATTATAAACATGTGGCAAGGAGTAGGACAAGCAATGTA

TGCCCCCCCCATCAGAGGAATAATTAAATGTGCATCAAATATTACAGGAATACTATTGAC

AAGAGATGGTGGTAGAAATGCTAGCGAGCCTGAGACTTTTAGACCTGGAGGAGGAA

>TJ20170317_11

AGAAGAAGAGATAATAATCAGATCTGAAAATCTCACAAACAATGCCAAAACCATAATAGT

GCACCTTAATGACTCTGTAGAAATCAGTTGTACCAGACCCTCCAATAATACAATAACAAG

CATAAATATAGGACCAGGACGAGCATTCTATAGAACAGGAGACATAGTAGGAGATATAAG

ACAAGCCTTTTGTGAAATTAATGGAGCAAAATGGAACAAAACTTTAGGACAGGTAGCTAA

AAAATTAAAAGAGCACTTTAATAAGACAATAGAATTCCAGCCGCCCTCAGGGGGAGATCT

AGAAATTACAATGCATCATTTTAATTGTAGAGGGGAATTTTTCTATTGCAATACAACAAA

GCTGTTCAATAGTACTTGGATAGGAAATGAGACCATAAGGGAAGGTAATGACACTATCAT

ACTTCCATGCAGGATAAAGCAAATTATAAACATGTGGCAAGGAGTAGGACAAGCAATGTA

TGCCCCCCCCATCAGAGGAATAATTAAATGTGCATCAAATATTACAGGAATACTATTGAC

AAGAGATGGTGGTAGAAATGCTAGCGAGCCTGAGACTTTTAGACCTGGAGGAGGAA

>TJ20170315_1

AGAAGAAGAAATAATAATCAGATCTGAGAATCTCACAAACAATGCCAAAAACATAATAGT

ACACTTTAATGAATCTGTAGAAATCAATTGTACCAGACCCTCCAACAATACAAGAACAAG

TATAAGAATAGGACCAGGCCAAATGTTCTATAGAACAGGAGACATAATAGGAGATATAAG

GAAAGCACATTGTAATATTAGTGGAACAAAATGGAATGAAACTTTAAATAAGGTAACTGA

AAAATTAAAGGAGCACTTTAATAAGACAATAGTCTTTAAACCACCCTCAGGAGGAGATCT

AGAAACTACAATGTTTCATTTTAATTGTAGAGGGGAATTTTTCTATTGCAATACAACACA

ATTGTTTAATAGTGCA------GAAAATGGAACCAGGGAGGGGTCT------AATATCAC

ACTCCCATGTAGGATAAAACAAATTGTAAACATGTGGCAGGGAGTAGGACAAGCAATGTA

TGCTCCTCCCATCAAGGGAATAATTGAGTGTAGATCAAATATAACAGGAATAATATTAAC

AAGAGATGGTGGT---ACTGATGCAACAAATGAAACCTTCAGACCGGGAGGAGGAA

>TJ20170315_2

AGAAGAAGAGATAATAATCAGATCTGAAAATCTCACAAACAATGCCAAAACCATAATAGT

GCACCTTAATGAATCTGTAGAAATCAGTTGTACCAGACCCTCCAATAATACAATAACAAG

CATAAATATAGGACCAGGACGAGCATTCTATAGAACAGGAGACATAATAGGAGATATAAG

ACAAGCCTTTTGTAAAATTAATGGAACAAAATGGAACAAAACTTTAGGACAGGTAGCTAA

AAAATTAAAAGAGCACTTTAATAAGACAATAGAATTCCAGCCGCCCTCAGGGGGAGATCT

AGAAATTACAATGCATCATTTTAATTGTAGAGGGGAATTTTTCTATTGCAATACAACAAA

GCTGTTCAATAGTACTTGGATAGGAAATGAGACCATAGGGGAAGGTAATGACACTATCAT

ACTTCCATGCAGGATAAAGCAAATTATAAACATGTGGCAAGGAGTAGGACAAGCAATGTA

TGCTCCCCCCATCAGAGGAATAATTAAATGTGCATCAAATATTACAGGAATACTATTGAC

AAGAGATGGTGGTAGAAATGCTAGC------GAGACTTTTAAACCTGGAGGAGGAA

>TJ20170315_3

AGAAGAAGAGATAATAATCAGATCTGAAAATCTCACAAACAATGCCAAAACCATAATAGT

GCACCTTAATGAAGCTGTAGAAATCAGTTGTACCAGACCCTCCAATAATACAATAACAAG

CATAAATATAGGACCAGGACGAGCATTCTATAGAACAGGAGACATAATAGGAGATATAAG

ACAAGCCTTTTGTGAAATTAATGGAACAAAATGGAACAAAACTTTAGGACAGGTAGCTAA

AAAATTAAAAGAGCACTTTAATAAGACAATAGAATTCCAGCCGCCCTCAGGGGGAGATCT

AGAAATTACAATGCATCATTTTAATTGTAGAGGGGAATTTTTCTATTGCAATACAACAAA

GCTGTTCAATAGTACTTGGATAGGAAATGAGACCATAGGGGAAGGTAATGACACTATCAT

ACTTCCATGCAGGATAAAGCAAATTATAAACATGTGGCAAGGAGTAGGACAAGCAATGTA

TGCTCCCCCCATCAGAGGAATAATTAAATGTGCATCAAATATTACAGGAATACTATTGAC

AAGAGATGGTGGTAGAAATGCTAGC------GAGACTTTTAGACCTGGAGGAGGAA

>TJ20170315_4

AGAAGAAGAAATAATAATCAGATCTGAGAATCTCACAAACAATGCCAAAAACATAATAGT

ACACTTTAATGAATCTGTAGAAATCAATTGTACCAGACCCTCCAACAATACAAGAACAAG

TATAAGAATAGGACCAGGCCAAATGTTCTATAGAACAGGAGACATAATAGGAGATATAAG

GAAAGCACATTGTAATATTAGTGGAACAAAATGGAATGAAACTTTAAATAAGGTAACTGA

AAAATTAAAGGAGCACTTTAATAAGACAATAGTCTTTAAACCACCCTCAGGAGGAGATCT

AGAAACTACAATGTTTCATTTTAATTGTAGAGGGGAATTTTTCTATTGCAATACAACACA

ATTGTTTAATAGTGCA------GAAAATGGAACCAGGGAGGGGTCT------AATATCAC

ACTCCCATGTAGGATAAAACAAATTGTAAACATGTGGCAGGGAGTAGGACAAGCAATGTA

TGCTCCTCCCATCAAGGGAATAATTGAGTGTAGATCAAATATAACAGGAATAATATTAAC

AAGAGATGGTGGT---ACTGATGCAACAAATGAAACCTTCAGACCGGGAGGAGGAA

>TJ20170315_5

AGAAGAAGAAATAATAATCAGATCTGAGAATCTCACAAACAATGCCAAAAACATAATAGT

ACACTTTAATGAATCTGTAGAAATCAATTGTACCAGACCCTCCAACAATACAAGAACAAG

TATAAGAATAGGACCAGGCCAAATGTTCTATAGAACAGGAGACATAATAGGAGATATAAG

GAAAGCACATTGTAATATTAGTGGAACAAAATGGAATGAAACTTTAAATAAGGTAACTGA

AAAATTAAAGGAGCACTTTAATAAGACAATAGTCTTTAAACCACCCTCAGGAGGAGATCT

AGAAACTACAATGTTTCATTTTAATTGTAGAGGGGAATTTTTCTATTGCAATACAACACA

ATTGTTTAATAGTGCA------GAAAATGGAACCAGGGAGGGGTCT------AATATCAC

ACTCCCATGTAGGATAAAACAAATTGTAAACATGTGGCAGGGAGTAGGACAAGCAATGTA

TGCTCCTCCCATCAAGGGAATAATTGAGTGTAGATCAAATATAACAGGAATAATATTAAC

AAGAGATGGTGGT---ACTGATGCAACAAATGAAACCTTCAGACCGGGAGGAGGAA

>TJ20170315_6

AGAAGAAGAGATAATAATCAGATCTGAAGATCTCACAAACAATGCCAAAACCATAATAGT

GCACCTTAATGAATCTGTAGAAATCAGTTGTACCAGACCCTCCAATAATACAATAACAAG

CATAAATATAGGACCAGGACGAGCATTCTATAGAACAGGAGACATAATAGGAGATATAAG

ACAAGCCTTTTGTAAAATTAATGGAACAAAATGGAACAAAACTTTAGGACAGGTAGCTAA

AAAATTAAAAGAGCACTTTAATAAGACAATAATATTCCAGCCGCCCTCAGGGGGAGATCT

AGAAATTACAATGCATCATTTTAATTGTAGAGGGGAATTTTTCTATTGCAATACAACAAA

GCTGTTCAATAGTACTTGGATAGGAAATGAGACCATAGAGGAAGGTAATGACACTATCAT

ACTTCCATGCAGGATAAAGCAAATTATAAACATGTGGCAAGGAGTAGGACAAGCAATGTA

TGCTCCCCCCATCAGAGGAATAATTAAATGTGCATCAAATATTACAGGAATACTATTGAC

AAGAGATGGTGGTGGAAATGCTAGCGAGTCTGAGACTTTTAGACCTGGAGGAGAA-

>TJ20170315_7

AGAAGAAGAAATAATAATCAGATCTGAGAATCTCACAAACAATGCCAAAAACATAATAGT

ACACTTTAATGAATCTGTAGAAATCAATTGTACCAGACCCTCCAACAATACAAGAACAAG

TATAAGAATAGGACCAGGCCAAATGTTCTATAGAACAGGAGACATAATAGGAGATATAAG

GAAAGCACATTGTAATATTAGTGGAACAAAATGGAATGAAACTTTAAATAAGGTAACTGA

AAAATTAAAGGAGCACTTTAATAAGACAATAGTCTTTAAACCACCCTCAGGAGGAGATCT

AGAAACTACAATGTTTCATTTTAATTGTAGAGGGGAATTTTTCTATTGCAATACAACACA

ATTGTTTAATAGTGCA------GAAAATGGAACCAGGGAGGGGTCT------AATATCAC

ACTCCCATGTAGGATAAAACAAATTGTAAACATGTGGCAGGGAGTAGGACAAGCAATGTA

TGCTCCTCCCATCAAGGGAATAATTGAGTGTAGATCAAATATAACAGGAATAATATTAAC

AAGAGATGGTGGT---ACTGATGCAACAAATGAAACCTTCAGACCGGGAGGAGGAA

>TJ20170315_8

AGAAGAAGAGATAATAATCAGATCTGAGAATCTCACAAACAATGCCAAAAACATAATAGT

GCACTTTAATGAATCTGTAGAAATCAATTGTATCAGACCCTCCAACAATACAAGAACAAG

TATAAGAATAGGACCAGGCCAAGTGTTCTATAGAACAGGAGACATAATAGGAGATATAAG

GAAAGCACATTGTAATATTAATGGAACAAAATGGAATGAAACTTTAAATAAGGTAACTGA

AAAATTAAAAGAGCACTTTAATAGGACAATAGTCTTTCAACCACCCTCAGGAGGAGATCT

AGAAACTACAATGTTTCATTTCAATTGTAGAGGGGAATTTTTCTATTGCAATACAACACA

ATTGTTTAATAGTGCA------GAAAATGGAACCAGGGAGGGGGAT------AATATCAC

ACTCCCATGTAGGATAAAACAAATTGTAAACATGTGGCAGGGAGTAGGACAAGCAATGTA

TGCTCCTCCTATCAAGGGAATAATTGAGTGTAGATCAAATATAACAGGAATAATATTAAC

AAGAGATGGTGGT---ACTGATGCAACAAATGACACCTTCAGACCGGGAGGAGGAA

>TJ20170315_9

AGAAGAAGAGATAATAATCAGATCTGAAAATCTCACAAACAATGCCAAAACCATAATAGT

GCACCTTAATGAATCTGTAGAAATCAGTTGTACCAGACCCTCCAATAATACAATAACAAG

CATAAATATAGGACCAGGACGAGCATTCTATAGAACAGGAGACATAATAGGAGATATAAG

ACAAGCCTTTTGTGAAATTAATGGAGCAAAATGGAACAAAACTTTAGGACAGGTAGCTAA

AAAATTAAAAGAGCACTTTAATAGGACAATAGAATTCCAGCCGCCCTCAGGGGGAGATCT

AGAAATTACAATGCATCATTTTAATTGTAGAGGGGAATTTTTCTATTGCAATACAACAAA

GCTGTTCAATAGTACTTGGATAGGAAATGAGACCATAGGGAAAGGTAATGACACTATCAT

ACTTCCATGCAGGATAAAGCAAATTATAAACATGTGGCAAGGAGTAGGACAAGCAATGTA

TGCTCCCCCCATCAGAGGAATAATTAAATGTGCATCAAATATTACAGGAATACTATTGAC

AAGAGATGGTGGTGGAAATGATAGCAAGCCTGAGATTTTTAGACCTGGAGGAGGAA

>TJ20170315_10

AGAAGAAGAGATAATAATCAGATCTGAAAATCTCACAAACAATGCCAAAACCATAATAGT

GCACCTTAATGAATCTGTAGAAATCAGTTGTACCAGACCCTCCAATAATACAATAACAAG

CATAAATATAGGACCAGGACGAGCATTCTATAGAACAGGAGACATAATAGGAGACATAAG

ACAAGCCTTTTGTGAAATTAATGGAGCAAAATGGAACAAAACTTTAGGACAGGTAGCTAA

AAAATTAAAAGAGCACTTTAATAGGACAATAGAATTCCAGCCGCCCTCAGGGGGAGATCT

AGAAATTACAATGCATCATTTTAATTGTAGAGGGGAATTTTTCTATTGCAATACAACAAA

GCTGTTCAATAGTACTTGGATAGGAAATGAGACCATAAGGGAAGGTAATGACACTATCAT

ACTTCCATGCAGGATAAAGCAAATTATAAACATGTGGCAAGGAGTAGGACAAGCAATGTA

TGCTCCCCCCATCAGAGGAATAATTAAATGTGCATCAAATATTACAGGAATACTATTGAC

AAGAGATGGTGGTGGAAATGATAGCAAGCCTGAGACTTTTAGACCTGGAGGAGGAA

>TJ20170315_11

AGAAGAAGAAATAATAATCAGATCTGAGAATCTCACAAACAATGCCAAAAACATAATAGT

ACACTTTAATGAATCTGTAGAAATCAATTGTACCAGACCCTCCAACAATACAAGAACAAG

TATAAGAATAGGACCAGGCCAAATGTTCTATAGAACAGGAGACATAATAGGAGATATAAG

GAAAGCACATTGTAATATTAGTGGAACAAAATGGAATGAAACTTTAAATAAGGTAACTGA

AAAATTAAAGGAGCACTTTAATAAGACAATAGTCTTTAAACCACCCTCAGGAGGAGATCT

AGAAACTACAATGTTTCATTTTAATTGTAGAGGGGAATTTTTCTATTGCAATACAACACA

ATTGTTTAATAGTGCA------GAAAATGGAACCAGGGAGGGGTCT------AATATCAC

ACTCCCATGTAGGATAAAACAAATTGTAAACATGTGGCAGGGAGTAGGACAAGCAATGTA

TGCTCCTCCCATCAAGGGAATAATTGAGTGTAGATCAAATATAACAGGAATAATATTAAC

AAGAGATGGTGGT---ACTGATGCAACAAATGAAACCTTCAGACCGGGAGGAGGAA

>TJ20170315_12

AGAAGAAGAGATAATAATCAGATCTGAAAATATCACAAACAATGCCAAAACCATAATAGT

GCACCTTAATGAATCTGTAGAAATCAGTTGTACCAGACCCTCCAATAATACAATAACAAG

CATAAATATAGGACCAGGACGAGCATTCTATAGAACAGGAGACATAATAGGAGATATAAG

ACAAGCCTTTTGTGAAATTAATGGAACAAAATGGAACAAAACTTTAGGACAGGTAGCTAA

AAAATTAAAAGAGCACTTTAATAAGACAATAGAATTCCAGCCGCCCTCAGGGGGAGATCT

AGAAATTACAATGCATCATTTTAATTGTAGAGGGGAATTTTTCTATTGCAATACAACAAA

GCTGTTCAATAGTACTTGGATAGGAAATGAGACCATAGGGGAAGGTAATGACACTATCAT

ACTTCCATGCAGGATAAAGCAAATTATAAACATGTGGCAAGGAGTAGGACAAGCAATGTA

TGCTCCCCCCATCAGAGGAATAATTAAATGTGCATCAAATATTACAGGAATACTATTGAC

AAGAGATGGTGGAAGAAATGCTAGCGAGCCTGAGAC-TTTAGACCTGGAGGAGGAA

>TJ20170315_13

AGAAGAAGAGATAATAATCAGATCTGAAAATCTCACAAACAATGCCAAAACCATAATAGT

GCACCTTAATGAATCTGTAGAAATCAGTTGTACCAGACCCTCCAATAATACAATAACAAG

CATAAATATAGGACCAGGACGAGCATTCTATAGAACAGGAGACATAATAGGAGATATAAG

ACAAGCCTTTTGTAAAATTAATGGAACAAAATGGAACAAAACTTTAGGACAGGTAGCTAA

AAAATTAAAAGAGCACTTTAATAAGACAATAAAATTCCAGCCGCCCTCAGGGGGAGATCT

AGAAATTACAATGCATCATTTTAATTGTAGAGGGGAATTTTTCTATTGCAATACAACAAA

GCTGTTCAATAGTACTTGGATAGGAAATGAGACCATAGAGGAAGGTAATGACACTATCAT

ACTTCCATGCAGGATAAAGCAAATTATAAACATGTGGCAAGGAGTAGGACAAGCAATGTA

TGCTCCCCCCATCAGAGGAATAATTAAATGTGCATCAAATATTACAGGAATACTATTGAC

AAGAGATGGTGGTGGAAATGATAGCGAGACTGAGACTTTTAGACCTGGAGGAGGAA

>TJ20170315_14

AGAAGAAGAAATAATAATCAGATCTGAGAATCTCACAAACAATGCCAAAAACATAATAGT

ACACTTTAATGAATCTGTAGAAATCAATTGTACCAGACCCTCCAACAATACAAGAACAAG

TATAAGAATAGGACCAGGCCAAATGTTCTATAGAACAGGAGACATAATAGGAGATATAAG

GAAAGCACATTGTAATATTAGTGGAACAAAATGGAATGAAACTTTAAATAAGGTAACTGA

AAAATTAAAGGAGCACTTTAATAAGACAATAGTCTTTAAACCACCCTCAGGAGGAGATCT

AGAAACTACAATGTTTCATTTTAATTGTAGAGGGGAATTTTTCTATTGCAATACAACACA

ATTGTTTAATAGTGCA------GAAAATGGAACCAGGGAGGGGTCT------AATATCAC

ACTCCCATGTAGGATAAAACAAATTGTAAACATGTGGCAGGGAGTAGGACAAGCAATGTA

TGCTCCTCCCATCAAGGGAATAATTGAGTGTAGATCAAATATAACAGGAATAATATTAAC

AAGAGATGGTGGT---ACTGATGCAACAAATGAAACCTTCAGACCGGGAGGAGGAA

>TJ20170315_15

------------AATAATCAGATCTGAAAATCTCACAAACAATGCCAAAACCATAATAGT

GCACCTTAATGAATCTGTAGAAATCAGTTGTACCAGACCCTCCAATAATACAAGAACAAG

CATAAATATAGGACCAGGACAAGCATTCTATAGAACAGGAGACATAATAGGAGATATAAG

ACAAGCCTTTTGTAAAATTAATGGAACAAAATGGAACAAAACTTTAGGACAGGTAGCTAA

AAAATTAAAAGAGCACTTTAATAAGCCAATAAAATTCCAGCCGCCCTCAGGGGGAGATCT

AGAAATTACAATGCATCATTTTAATTGTAGAGGGGAATTTTTCTATTGCAATACAACAAA

GCTGTTCAATAGTACTTGGATAGGAAATGAGACCATAGGGAAAGGTAATGACACTATCAT

ACTTCCATGCAGGATAAAGCAAATTATAAACATGTGGCAAGGAGTAGGACAAGCAATGTA

TGCTCCCCCCATCAGAGGAATAATTAAATGTGCATCAAATATTACAGGAATACTATTGAC

AAGAGATGGTGGT------GATAACGAGTCTGAGACTTTTA---------------

>TJ20170316_1

AGAAGAAGAGATAATAATCAGATCTGAAAATCTCACAAACAATGCCAAAACCATAATAGT

GCACCTTAATGAATCTGTAGAAATCAGTTGTACCAGACCCTCCAATAATACAATAACAAG

CATAAATATAGGACCAGGACGAGCATTCTATAGAACAGGAGACATAGTAGGAGATATAAG

ACAAGCCTTTTGTGAAATTAATGGAGCAAAATGGAACAAAACTTTAGGACAGGTAGCTAA

AAAATTAAAAGAGCACTTTAATAAGACAATAGAATTCCAGCCGCCCTCAGGGGGAGATCT

AGAAATTACAATGCATCATTTTAATTGTAGAGGGGAATTTTTCTATTGCAATACAACAAA

GCTGTTCAATAGTACTTGGATAGGAAATGAGACCATAGGGGAAGGTAATGACACTATCAT

ACTTCCATGCAGGATAAAGCAAATTATAAACATGTGGCAAGGAGTAGGACAAGCAATGTA

TGCTCCCCCCATCAGAGGAATAATTAAATGTGCATCAAATATTACAGGAATACTATTGAC

AAGAGATGGTGGTAGAAATGCTAGCGAGCGTGAGACTTTTAGACCTGGAGGAGGAA

>TJ20170316_2

AGAAGAAGAGAAAATAATCAGATCTGAAAATCTCACAAACAATGCCAAAACCATAATAGT

GCACCTTAATGAATCTGTAGAAATCAGTTGTACCAGACCCTCCAATAATACAATAACAAG

CATAAATATAGGACCAGGACGAGCATTCTATAGAACAGGAGACATAGTAGGAGATATAAG

ACAAGCCTTTTGTGAAATTAATGGAGCAAAATGGAACAAAACTTTAGGACAGGTAGCTAA

AAAATTAAAAGAGCACTTTAATAAGACAATAGAATTCCAGCCGCCCTCAGGGGGAGATCT

AGAAATTACAATGCATCATTTTAATTGTAGAGGGGAATTTTTCTATTGCAATACAACAAA

GCTGTTCAATAGTACTTGGATAGGAAATGAGACCATAGGGGAAGGTAATGACACTATCAT

ACTTCCATGCAGGATAAAGCAAATTATAAACATGTGGCAAGGAGTAGGACAAGCAATGTA

TGCTCCCCCCATCAGAGGAATAATTAAATGTGCATCAAATATTACAGGAATACTATTGAC

AAGAGATGGTGGTAGAAATGCTAGCGAGCCTGAGACTTTTAGACCTGGAGGAGGAA

>TJ20170316_3

AGAAGAAGAGATAATAATCAGATCTGAAAATCTCACAAACAATGCCAAAACCATAATAGT

GCACCTTAATGAATCTGTAGAAATCAGTTGTACCAGACCCTCCAATAATACAATAACAAG

CATAAATATAGGACCAGGACGAGCATTCTATAGAACAGGAGACATAGTAGGAGATATAAG

ACAAGCCTTTTGTGAAATTAATGGAGCAAAATGGAACAAAACTTTAGGACAGGTAGCTAA

AAAATTAAAAGAGCACTTTAATAAGACAATAGAATTCCAGCCGCCCTCAGGGGGAGATCT

AGAAATTACAATGCATCATTTTAATTGTAGAGGGGAATTTTTCTATTGCAATACAACAAA

GCTGTTCAATAGTACTTGGATAGGAAATGAGACCATAGGGGAAGGTAATGACACTATCAT

ACTTCCATGCAGGATAAAGCAAATTATAAACATGTGGCAAGGAGTAGGACAAGCAATGTA

TGCTCCCCCCATCAGAGGAATAATTAAATGTGCATCAAATATTACAGGAATACTATTGAC

AAGAGATGGTGGTAGAAATGCTAGCGAGCCTGAGACTTTTAGACCTGGAGGAGGAA

>TJ20170316_4

AGAAGAAGAGATAATAATCAGATCTGAAAATCTCACAAACAATGCCAAAACCATAATAGT

GCACCTTAATGAATCTGTAGAAATCAGTTGTACCAGACCCTCCAATAATACAATAACAAG

CATAAATATAGGACCAGGACGAGCATTCTATAGAACAGGAGACATAGTAGGAGATATAAG

ACAAGCCTTTTGTGAAATTAATGGAGCAAAATGGAACAAAACTTTAGGACAGGTAGCTAA

AAAATTAAAAGAGCACTTTAATAAGACAATAGAATTCCAGCCGCCCTCAGGGGGAGATCT

AGAAATTACAATGCATCATTTTAATTGTAGAGGGGAATTTTTCTATTGCAATACAACAAA

GCTGTTCAATAGTACTTGGATAGGAAATGAGACCATAGGGGAAGGTAATGACACTATCAT

ACTTCCATGCAGGATAAAGCAAATTATAAACATGTGGCAAGGAGTAGGACAAGCAATGTA

TGCTCCCCCCATCAGAGGAATAATTAAATGTGCATCAAATATTACAGGAATACTATTGAC

AAGAGATGGTGGTAGAAATGCTAGCGAGCCTGAGACTTTTAGACCTGGAGGAGGAA

>TJ20170316_5

AGAAGAAGAGATAATAATCAGATCTGAAAATCTCACAAACAATGCCAAAACCATAATAGT

GCACCTTAATGAATCTGTAGAAATCAGTTGTACCAGACCCTCCAATAATACAATAACAAG

CATAAATATAGGACCAGGACGAGCATTCTATAGAACAGGAGACATAGTAGGAGATATAAG

ACAAGCCTTTTGTGAAATTAATGGAGCAAAATGGAACAAAACTTTAGGACAGGTAGCTAA

AAAATTAAAAGAGCACTTTAATAAGACAATAGAATTCCAGCCGCCCTCAGGGGGAGATCT

AGAAATTACAATGCATCATTTTAATTGTAGAGGGGAATTTTTCTATTGCAATACAACAAA

GCTGTTCAATAGTACTTGGATAGGAAATGAGACCATAGGGGAAGGTAATGACACTATCAT

ACTTCCATGCAGGATAAAGCAAATTATAAACATGTGGCAAGGAGTAGGACAAGCAATGTA

TGCTCCCCCCATCAGAGGAATAATTAAATGTGCATCAAATATTACAGGAATACTATTGAC

AAGAGATGGTGGTAGAAATGCTAGCGAGCTTGAGACTTTTAGACCTGGAGGAGGAA

>TJ20170316_6

AGAAGAAGAGATAATAATCAGATCTGAAAATCTCACAAACAATGCCAAAACCATAATAGT

GCACCTTAATGAATCTGTAGAAATCAGTTGTACCAGACCCTCCAATAATACAATAACAAG

CATAAATATAGGACCAGGACGAGCATTCTATAGAACAGGAGACATAGTAGGAGATATAAG

ACAAGCCTTTTGTGAAATTAATGGAGCAAAATGGAACAAAACTTTAGGACAGGTAGCTAA

AAAATTAAAAGAGCACTTTAATAAGACAATAGAATTCCAGCCGCCCTCAGGGGGAGATCT

AGAAATTACAATGCATCATTTTAATTGTAGAGGGGAATTTTTCTATTGCAATACAACAAA

GCTGTTCAATAGTACTTGGATAGGAAATGAGACCATAGGGGAAGGTAATGACACTATCAT

ACTTCCATGCAGGATAAAGCAAATTATAAACATGTGGCAAGGAGTAGGACAAGCAATGTA

TGCTCCCCCCATCAGAGGAATAATTAAATGTGCATCAAATATTACAGGAATACTATTGAC

AAGAGATGGTGGTAGAAATGCTAGCGAGCCTGAGACTTTTAGACCTGGAGGAGGAA

>TJ20170316_7

AGAAGAAGAGATAATAATCAGATCTGAAAATCTCACAAACAATGCCAAAACCATAATAGT

GCACCTTAATGAATCTGTAGAAATCAGTTGTACCAGACCCTCCAATAATACAATAACAAG

CATAAATATAGGACCAGGACGAGCATTCTATAGAACAGGAGACATAGTAGGAGATATAAG

ACAAGCCTTTTGTGAAATTAATGGAGCAAAATGGAACAAAACTTTAGGACAGGTAGCTAA

AAAATTAAAAGAGCACTTTAATAAGACAATAGAATTCCAGCCGCCCTCAGGGGGAGATCT

AGAAATTACAATGCATCATTTTAATTGTAGAGGGGAATTTTTCTATTGCAATACAACAAA

GCTGTTCAATAGTACTTGGATAGGAAATGAGACCATAGGGGAAGGTAATGACACTATCAT

ACTTCCATGCAGGATAAAGCAAATTATAAACATGTGGCAAGGAGTAGGACAAGCAATGTA

TGCTCCCCCCATCAGAGGAATAATTAAATGTGCATCAAATATTACAGGAATACTATTGAC

AAGAGATGGTGGTAGAAATGCTAGCGAGCCTGAGACTTTTAGACCTGGAGGAGGAA

>TJ20170316_8

AGAAGAAGAGATAATAATCAGATCTGAAAATCTCACAAACAATGCCAAAACCATAATAGT

GCACCTTAATGAATCTGTAGAAATCAGTTGTACCAGACCCTCCAATAATACAATAACAAG

CATAAATATAGGACCAGGACGAGCATTCTATAGAACAGGAGACATAGTAGGAGATATAAG

ACAAGCCTTTTGTGAAATTAATGGAGCAAAATGGAACAAAACTTTAGGACAGGTAGCTAA

AAAATTAAAAGAGCACTTTAATAAGACAATAGAATTCCAGCCGCCCTCAGGGGGAGATCT

AGAAATTACAATGCATCATTTTAATTGTAGAGGGGAATTTTTCTATTGCAATACAACAAA

GCTGTTCAATAGTACTTGGATAGGAAATGAGACCATAGGGGAAGGTAATGACACTATCAT

ACTTCCATGCAGGATAAAGCAAATTATAAACATGTGGCAAGGAGTAGGACAAGCAATGTA

TGCTCCCCCCATCAGAGGAATAATTAAATGTGCATCAAATATTACAGGAATACTATTGAC

AAGAGATGGTGGTAGAAATGCTAGCGAGCTTGAGACTTTTAGACCTGGAGGAGGAA

>TJ20170316_9

AGAAAAAGAGATAATAATCAGATCTGAAAATCTCACAAACAATGCCAAAACCATAATAGT

GCACCTTAATGAATCTGTAGAAATCAGTTGTACCAGACCCTCCAATAATACAATAACAAG

CATAAATATAGGACCAGGACGAGCATTCTATAGAACAGGAGACATAGTAGGAGATATAAG

ACAAGCCTTTTGTGAAATTAATGGAGCAAAATGGAACAAAACTTTAGGACAGGTAGCTAA

AAAATTAAAAGAGCACTTTAATAAGACAATAGAATTCCAGCCGCCCTCAGGGGGAGATCT

AGAAATTACAATGCATCATTTTAATTGTAGAGGGGAATTTTTCTATTGCAATACAACAAA

GCTGTTCAATAGTACTTGGATAGGAAATGAGACCATAAGGGAAGGTAATGACACTATCAT

ACTTCCATGCAGGATAAAGCAAATTATAAACATGTGGCAAGGAGTAGGACAAGCAATGTA

TGCTCCCCCCATCAGAGGAATAATTAAATGTGCATCAAATATTACAGGAATACTATTGAC

AAGAGATGGTGGTAAAAATGCTAGCGAGCCTGAGACTTTTAGACCTGGAGGAGGAA

>TJ20170316_10

AGAAGAAGAGATAATAATCAGATCTGAAAATCTCACAAACAATGCCAAAACCATAATAGT

GCACCTTAATGAATCTGTAGAAATCAGTTGTACCAGACCCTCCAATAATACAATAACAAG

CATAAATATAGGACCAGGACGAGCATTCTATAGAACAGGAGACATAGTAGGAGATATAAG

ACAAGCCTTTTGTGAAATTAATGGAGCAAAATGGAACAAAACTTTAGGACAGGTAGCTAA

AAAATTAAAAGAGCACTTTAATAAGACAATAGAATTCCAGCCGCCCTCAGGGGGAGATCT

AGAAATTACAATGCATCATTTTAATTGTAGAGGGGAATTTTTCTATTGCAATACAACAAA

GCTGTTCAATAGTACTTGGATAGGAAATGAGACCATAGGGGAAGGTAATGACACTATCAT

ACTTCCATGCAGGATAAAGCAAATTATAAACATGTGGCAAGGAGTAGGACAAGCAATGTA

TGCTCCCCCCATCAGAGGAATAATTAAATGTGCATCAAATATTACAGGAATACTATTGAC

AAGAGATGGTGGTAGAAATGCTAGCGAGCTTGAGACTTTTAGACCTGGAGGAGGAA

>TJ20170316_11

AGAAGAAGAGATAATAATCAGATCTGAAAATCTCACAAACAATGCCAAAACCATAATAGT

GCACCTTAATGAATCTGTAGAAATCAGTTGTACCAGACCCTCCAATAATACAATAACAAG

CATAAATATAAGACCAGGACGAGCATTCTATAGAACAGGAGACATAGTAGGAGATATAAG

ACAAGCCTTTTGTGAAATTAATAGAGCAAAATGGAACAAAACTTTAGGACAGGTAGCTAA

AAAATTAAAAGAGCACTTTAATAAGACAATAGAATTCCAGCCGCCCTCAGAGGGAGATCT

AGAAATTACAATGCATCATTTTAATTGTAGAGAGGAATTTTTCTATTGCAATACAACAAA

GCTGTTCAATAGTACTTAGATAGGAAATGAGACCATAAGGGAAAGTAATGACACTATCAT

ACTTCCATGCAGAATAAAGCAAATTATAAACATGTGGCAAAGAGTAGGACAAGCAATGTA

TGCTCCCCCCATCAGAGGAATAATTAAATGTGCATCAAATATTACAGGAATACTATTGAC

AAGAGATAGTAGTAGAAATGCTAGCGAGCCTGAGACTTTTAGACCTGGAAGAAGAA

>TJ20170316_12

AGAAGAAGAGATAATAATCAGATCTGAAAATCTCACAAACAATGCCAAAACCATAATAGT

GCACCTTAATGAATCTGTAGAAATCAGCTGTACCAGACCCTCCAATAATACAATAACAAG

CATAAATATAGGACCAGGACGAGCATTCTATAGAACAGGAGACATAGTAGGAGATATAAG

ACAAGCCTTTTGTGAAATTAATGGAGCAAAATGGAACAAAACTTTAGGACAGGTAGCTAA

AAAATTAAAAGAGCACTTTAATAAGACAATAGAATTCCAGCCGCCCTCAGGGGGAGATCT

AGAAATTACAATGCATCATTTTAATTGTAGAGGGGAATTTTTCTATTGCAATACAACAAA

GCTGTTCAATAGTACTTGGATAGGAAATGAGACCATAGGGGAAGGTAATGACACTATCAT

ACTTCCATGCAGGATAAAGCAAATTATAAACATGTGGCAAGGAGTAGGACAAGCAATGTA

TGCTCCCCCCATCAGAGGAATAATTAAATGTGCATCAAATATTACAGGAATACTATTGAC

AAGAGATGGTGGTAGAAATGCTAGCGAGCCTGAGACTTTTAGACCTGGAGGAGGAA

>TJ20170316_13

AGAAGAAGAGATAATAATCAGATCTGAAAATCTCACAAACAATGCCAAAACCATAATAGT

GCACCTTAATGAATCTGTAGAAATCAGTTGTACCAGACCCTCCAATAATACAATAACAAG

CATAAATATAGGACCAGGACGAGCATTCTATAGAACAGGAGACATAGTAGGAGATATAAG

ACAAGCCTGTTGTGAAATTAATGGAGCAAAATGGAACAAAACTTTAGGACAGGTAGCTAA

AAAATTAAAAGAGCACTTTAATAAGACAATAGAATTCCAGCCACCCTCAGGGGGAGATCT

AGAAATTACAATGCATCATTTTAATTGTAGAGGGGAATTTTTCTATTGCAATACAACAAA

GCTGTTCAATAGTACTTGGATAGGAAATGAGACCATAGGGGAAGGTAATGACACTATCAT

ACTTCCATGCAGGATAAAGCAAAGTATAAACATGTGGCAAGGAGTAGGACAAGCAATGTA

TGCTCCCCCCATCAGAGGAATAATTAAATGTGCATCAAATATTACAGGAATACTATTGAC

AAGAGATGGTGGTAGAAATGCTAGAGAGCTTGAGACTTTTAGACCTGGAGGAGGAA

>TJ20170316_14

AGAAGAAGAGATAATAATCAGATCTGAAAATCTCACAAACAATGCCAAAACCATAATAGT

GCACCTTAATGAATCTGTAGAAATCAGTTGTACCAGACCCTCCAATAATACAATAACAAG

CATAAATATAGGACCAGGACGAGCATTCTATAGAACAGGAGACATAGTAGGAGATATAAG

ACAAGCCTTTTGTGAAATTAATGGAGCAAAATGGAACAAAACTTTAGGACAGGTAGCTAA

AAAATTAAAAGAGCACTTTAATAAGACAATAGAATTCCAGCCGCCCTCAGGGGGAGATCT

AGAAATTACAATGCATCATTTTAATTGTAGAGGGGAATTTTTCTATTGCAATACAACAAA

GCTGTTCAATAGTACTTGGATAGGAAATGAGACCATAGGGGAAGGTAATGACACTATCAT

ACGTCCATGCAGGATAAAGCAAATTATAAACATGTGGCAAGGAGTAGGACAAGCAATGTA

TGCTCCCCCCATCAGAGGAATAATTAAATGTGCATCAAATATTACAGGAATACTATTGAC

AAGAGATGGTGGTAGAAATGCTAGCGAGCCTGAGACTTTTAGACCTGGAGGAGGAA

>TJ20170316_15

AGAAGAAGAGATAATAATCAGATCTGAAAATCTCACAAACAATGCCAAAACCATAATAGT

GCACCTTAATGAATCTGTAGAAATCAGTTGTACCAGACCCTCCAATAATACAATAACAAG

CATAAATATAGGACCAGGACGAGCATTCTATAGAACAGGAGACATAGTAGGAGATATAAG

ACAAGCCTTTTGTGAAATTAATGGAGCAAAATGGAACAAAACTTTAGGACAGGTAGCTAA

AAAATTAAAAGAGCACTTTAATAAGACAATAGAATTCCAGCCGCCCTCAGGGGGAGATCT

AGAAATTACAATGCATCATTTTAATTGTAGAGGGGAATTTTTCTATTGCAATACAACAAA

GCTGTTCAATAGTACTTGGATAGGAAATGAGACCATAGGGGAAGGTAATGACACTATCAT

ACTTCCATGCAGGATAAAGCAAATTATAAACATGTGGCAAGGAGTAGGACAAGCAATGTA

TGCTCCCCCCATCAGAGGAATAATTAAATGTGCATCAAATATTACAGGAATACTATTGAC

AAGAGATGGTGGTAGAAATGCTAGCGAGCCTGAGACTTTTAGACCTGGAGGAGGAA

>TJ20170316_16

AGAAGAAGAGATAATAATCAGATCTGAAAATCTCACAAACAATGCCAAAACCATAATAGT

GCACCTTAATGAATCTGTAGAAATCAGTTGTACCAGACCCTCCAATAATACAATAACAAG

CATAAATATAGGACCAGGACGAGCATTCTATAGAACAGGAGACATAGTAGGAGATATAAG

ACAAGCCTTTTGTGAAATTAATGGAGCAAAATGGAACAAAACTTTAGGACAGGTAGCAAA

AAAATTAAAAGAGCACTTTAATAAGACAATAGAATTCCAGCCGCCCTCAGGGGGAGATCT

AGAAATTACAATGCATCATTTTAATTGTAGAGGGGAATTTTTCTATTGCAATACAACAAA

GCTGTTCAATAGTACTTGGATAGGAAATGAGACCATAGGGGAAGGTAATGACACTATCAT

ACTTCCATGCAGGATAAAGCAAATTATAAACATGTGGCAAGGAGTAGGACAAGCAATGTA

TGCTCCCCCCATCAGAGGAATAATTAAATGTGCATCAAATATTACAGGAATACTATTGAC

AAGAGATGGTGGTAGAAATGCTAGCGAGCCTGAGACTTTTAGACCTGGAGGAGGAA
